# Evaluation of a multi-component mucoadhesive buccal patch: cytokine-associated inflammatory responses and tissue remodeling in experimental models

**DOI:** 10.3389/fimmu.2026.1847576

**Published:** 2026-05-28

**Authors:** Rekha Rani Kokkanti, Sandhiya Viswanathan, Nandita Parida, Soumyajit Biswas, Gajraj Singh Kushwaha, Pratap Kumar Deheri, Alok Kumar Panda, Abikshyeet Panda, Atul Anand Bajoria, Srinivas Patnaik

**Affiliations:** 1 School of Biotechnology, Kalinga Institute of Industrial Technology, (KIIT) Deemed to be University, Bhubaneswar, Odisha, India; 2 Technology Business Incubator, KIIT University, Bhubaneswar, Odisha, India; 3 School of Applied Sciences, Kalinga Institute of Industrial Technology, (KIIT) Deemed to be University, Bhubaneswar, Odisha, India; 4 Kalinga Institute of Dental Science, Kalinga Institute of Industrial Technology, (KIIT) Deemed to be University, Bhubaneswar, Odisha, India

**Keywords:** cytokines, extracellular matrix, fibrosis, inflammation, mucoadhesive buccal patch, oral squamous cell carcinoma, oral submucous fibrosis

## Abstract

**Introduction:**

Dysregulated cytokine signaling is implicated in persistent inflammation and fibrosis in oral submucous fibrosis (OSMF) and has been reported in association with pathways observed in oral squamous cell carcinoma (OSCC). This study investigated cytokine associated inflammatory and fibrotic responses following treatment with a mucoadhesive buccal patch containing isotretinoin, bromelain, and limonene.

**Methods:**

The formulation was evaluated using lipopolysaccharide (LPS) induced acute inflammatory in vitro models and a carbon tetrachloride (CCl_₄_) induced *in vivo* oral fibrotic model. Gene expression analysis was performed using qPCR to assess IL-6, TNF-α, MMP-2, IL-2, and TGF-β expression. Protein expression of COX-2 and CK17 was also examined. Bromelain activity within the formulation was evaluated using collagen degradation assays under different concentrations and incubation periods. Histological analysis and functional assessment of mouth opening were conducted in vivo to evaluate fibrotic alterations.

**Results:**

LPS stimulation induced upregulation of IL-6, TNF-α, and MMP-2, while treatment with the formulation was associated with reduced expression of these inflammatory markers. Altered IL-2 and TGF-β expression suggested modulation of immune regulatory and fibrosis-associated pathways. The in vitro model represented an acute inflammatory epithelial response and did not fully recapitulate the fibrotic pathology of OSMF. Protein analysis demonstrated reduced COX-2 expression and alterations in CK17 levels, consistent with changes in inflammatory and epithelial responses. Bromelain retained enzymatic activity within the formulation and demonstrated concentration and time dependent collagen degradation. In vivo treatment showed reduced collagen-associated staining, improved tissue architecture, and changes in mouth opening, indicating attenuation of fibrotic progression.

**Discussion:**

These findings provide preliminary observations on cytokine-associated responses under inflammatory and fibrotic conditions relevant to OSMF. Further studies are required to define underlying mechanisms and evaluate translational applicability.

## Graphical Abstract

**Figure:**
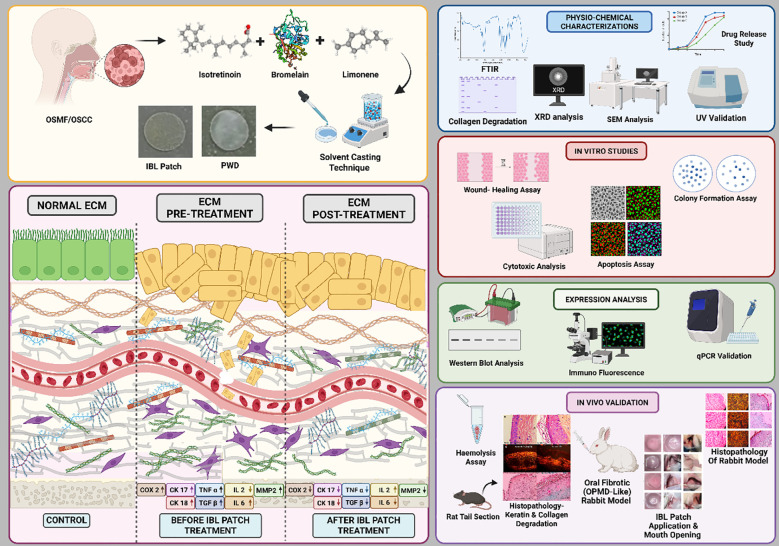

## Highlights

- Localized buccal delivery platform incorporating isotretinoin, bromelain, and limonene
- Altered expression of cytokine associated inflammatory markers (IL-6, TNF-α, TGF-β, IL2)
- Alteration of extracellular matrix related responses, including MMP-2 expression
- Effects observed in LPS induced acute inflammatory *in vitro* models
- Reduced collagen deposition and functional improvements in an *in vivo* chemically induced inflammation model

## 1 Introduction

Oral submucous fibrosis (OSMF) is a chronic inflammatory and fibrotic disorder of the oral mucosa characterized by progressive extracellular matrix (ECM) deposition and restricted tissue elasticity (1, 2). The condition is associated with persistent inflammation and an altered tissue microenvironment, which may contribute to disease progression. OSMF is also recognized for its potential association with the development of oral squamous cell carcinoma (OSCC), although the underlying mechanisms remain incompletely understood (3–5).

Cytokine signaling is recognized as an important component in inflammatory and fibrotic responses observed in OSMF. Pro-inflammatory cytokines such as IL-6 and TNF-α activate downstream signaling pathways, including NF-κB and MAPK, contributing to sustained inflammation and tissue remodeling. In parallel, TGF-β-mediated signaling is implicated in fibrosis through the promotion of extracellular matrix deposition and fibroblast activation (6, 7). Dysregulation of these pathways contributes to persistent inflammatory and fibrotic activity within the oral mucosal environment and may also influence epithelial and microenvironmental changes associated with tumor related processes (8). These observations suggest that OSMF and OSCC may share certain inflammatory features; however, they represent biologically distinct conditions with different cellular drivers.

Despite increasing understanding of cytokine-mediated inflammation, targeted modulation of cytokine signaling within the oral mucosal niche remains insufficiently explored. Current therapeutic approaches for OSMF are largely focused on symptomatic management and do not specifically address cytokine driven mechanisms underlying inflammation and fibrosis. This highlights the need for strategies that can influence these pathways within the local tissue environment.

Biomaterial based delivery systems, such as mucoadhesive buccal patches, provide a platform for localized intervention by enabling sustained contact with the oral mucosa. Multi-component formulations may offer the ability to influence multiple biological processes simultaneously, including inflammatory profiles, ECM remodeling, and epithelial responses (9–11). The formulation evaluated in this study integrates isotretinoin, bromelain, and limonene, each of which has been reported to influence inflammatory, ECM related, or epithelial associated processes relevant to the disease context. Isotretinoin is associated with regulation of epithelial differentiation, bromelain with proteolytic activity and ECM modulation, and limonene with anti-inflammatory effects (12–16). The combination of these components provides a basis for examining their collective influence on cytokine associated inflammatory pathways within the oral mucosal environment (17).

## 2 Materials and methods

### 2.1 Materials

Ethyl Cellulose (Sigma Aldrich, Cat# 46070), Dibutyl Phthalate 99% (Sigma Aldrich, Cat# 524980), Hydroxypropyl Methylcellulose (Sigma Aldrich, Cat# H7509), Sodium Alginate (Sigma Aldrich, Cat# W201502), Bromelain (Sigma Aldrich, Cat# B4882), Isotretinoin (Sigma Aldrich, Cat# PHR1188), Limonene (Sigma Aldrich, Cat# 183164), Sodium Carboxymethylcellulose (Sigma Aldrich, Cat# 419273) Artificial Saliva (Sigma Aldrich, Cat# SAE0149), Bromelain from pineapple stem (Jigs Chemical, Cat# 35079099), Isotretinoin (Jigs Chemical, Cat# 29362100), Limonene (Jigs Chemical, Cat# 29021900), Sodium bicarbonate (Sigma Aldrich, Cat # S5761-5006), Fetal bovine serum (Gibco, Cat # 10270-106), Penstrep Solution (Gibco, Cat # 15140), Dimethyl sulphoxide (DMSO) (Himedia, Cat # 67-68-5), MTT (1-(4,5-Dimethylthiazol-2-yl)-3,5-diphenylformazan) (Sigma Aldrich, Cat # M2003), Carbon tetrachloride (CCl4) (LOBA Chemi Pvt. Ltd, Cat # L50973Z2405), Ketamine Cat # KMV22401 and Xylazine Cat # FHK2003 (Indian immunological Ltd.), Lidocaine 10% Cat # KPNP48312 (Neon Laboratory Ltd.).

### 2.2 Standardization and optimization of mucoadhesive patch

**Figure 1:**
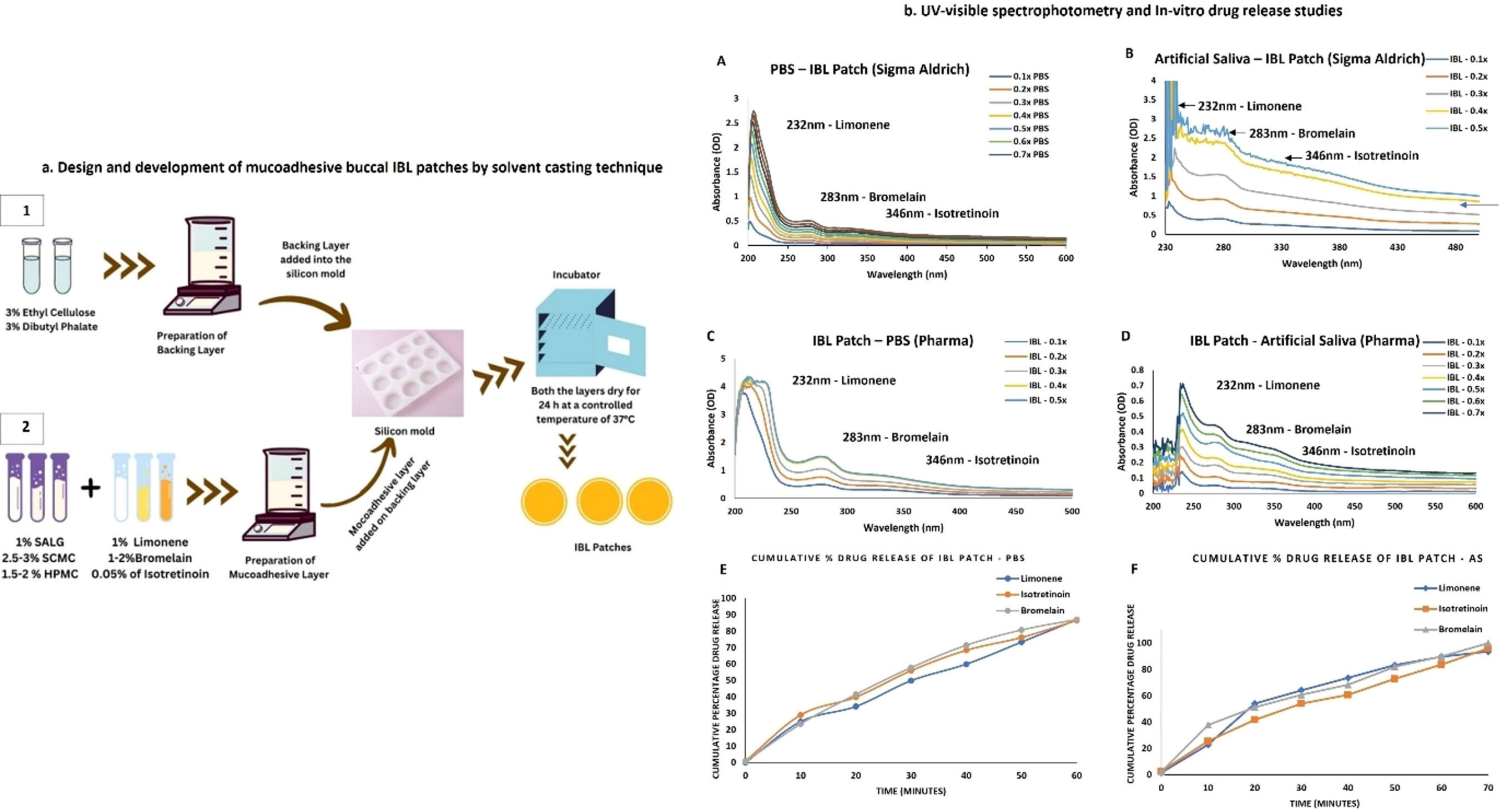
Preparation and *in vitro* release profile of the IBL buccal patch. **(a)** Schematic illustration of fabrication by solvent casting, showing preparation of the backing layer and IBL drugs loaded mucoadhesive layer, followed by assembly and drying. **(b)** UV–visible spectra of IBL patches prepared using Sigma-Aldrich grade drugs dissolved in **(A)** 1× PBS (pH 7.4) and **(B)** artificial saliva, and pharmaceutical-grade drugs dissolved in **(C)** 1× PBS (pH 7.4) and **(D)** artificial saliva. Absorption peaks at 232 nm (limonene), 283 nm (bromelain), and 346 nm (isotretinoin) are indicated. **(E)** Cumulative percentage release of isotretinoin, bromelain, and limonene in PBS (pH 7.4) over 60 min. **(F)** Cumulative percentage release in artificial saliva over 60 min. Data are presented as mean ± SD (n = 3).

The multi-component buccal patch was prepared using a solvent casting method with a bilayer design. The backing layer was formulated using ethyl cellulose (EC) and dibutyl phthalate (DBP) dissolved in acetone and cast onto a silicone mold, followed by controlled drying. The mucoadhesive layer was prepared by dissolving sodium alginate (SALG), sodium carboxymethyl cellulose (SCMC), and hydroxypropyl methylcellulose (HPMC) in an aqueous medium. Isotretinoin, bromelain, and limonene were incorporated into the polymeric solution under continuous stirring to ensure uniform dispersion. The resulting mixture was cast over the dried backing layer and allowed to dry under controlled conditions to obtain bilayer patches. The dried patches were carefully peeled and stored under appropriate conditions until further use. The composition was optimized to achieve suitable mechanical properties, mucoadhesion, and controlled release characteristics. Physicochemical and mucoadhesive evaluations are summarized in **Figure 1a**; **Table 1**. The concentrations of active components were selected based on preliminary optimization and reported biological activity ranges (**Supplementary Tables 1A**, **S1B**) (18).

**Table 1:** Different parameters of prepared buccal patches.

Formulation	Patch thickness (mm)	Weight variation (mg)	pH of patch dispersion	Folding endurance	Drug content uniformity (%)
F1	3.53 ± 0.15	143.23 ± 1.72	6.38 ± 0.02	195 ± 1.52	98.96 ± 0.49
F2	3.93 ± 0.05	167.86 ± 1.55	6.36 ± 0.02	179 ± 2.51	97.83 ± 0.60
F3	3.75 ± 0.05	145 ± 0.96	6.40 ± 0.01	197 ± 1.52	98 ± 0.2
F4	3.93 ± 0.05	166.56 ± 0.90	6.33 ± 0.03	224 ± 2.51	97.83 ± 0.60
F5	3.2 ± 0.1	136.6 ± 0.97	6.43 ± 0.05	192 ± 2	99.3 ± 0.36
F6	3.4 ± 0.1	133.9 ± 1.62	6.35 ± 0.06	183 ± 2.51	97.9 ± 0.81
F7	3.46 ± 0.08	127.1 ± 1.1	6.31 ± 0.05	257 ± 1.53	98.36 ± 0.92
F8	3.37 ± 0.15	129 ± 0.95	6.36 ± 0.05	203 ± 1	95.3 ± 0.87

Values represented as mean ± SD, (n=3), P < 0.05.

### 2.3 Characterization of IBL mucoadhesive buccal patches

#### 2.3.1 Physical appearance

The physical appearance of IBL loaded mucoadhesive buccal patches (MABPs) (n = 3) were evaluated visually, with attention to color, texture, and transparency.

#### 2.3.2 Thickness and weight uniformity

Film thickness was measured at five distinct locations on each IBL patch using a digital Vernier caliper (Yuri, India), and the mean thickness was calculated. For weight uniformity, five patches from each batch were individually weighed using a digital balance, and the average weight was determined.

#### 2.3.3 pH of patch dispersion

The pH of the patch dispersion was measured to estimate the local pH upon hydration and assess potential mucosal compatibility. Each IBL patch was cut into small pieces and dispersed in 5 mL of distilled water, followed by incubation for 10 minutes to allow hydration. The pH of the resulting dispersion was measured using a calibrated pH meter (Eutech, India).

#### 2.3.4 Folding endurance

Folding endurance was determined by repeatedly folding each patch at the same location until breakage occurred. The number of folds sustained before rupture was recorded as the folding endurance value. This procedure was performed manually.

#### 2.3.5 Drug content uniformity

Patches were finely cut, dissolved in 0.1N NaOH with magnetic stirring, and appropriately diluted. Drug content was quantified using a UV-spectrophotometer (Agilent Cary 600) within the 200–600 nm wavelength range. Drug content uniformity was calculated as:


$$\text{Drug content Uniformity }=\frac{\left(\text{Actual amount of drugs in patch}\right)}{\text{Theoretical amount of the drug in patch}}\times 100$$

#### 2.3.6 Swelling index

A 2% agar gel was cast onto a glass plate, and the initial weight (W1) of each patch was recorded. Patches were placed on the gel and incubated at 37 °C for 8 hours. At hourly intervals, patches were blotted to remove excess water and weighed (W2). The swelling index was determined as:


$$Swelling\;Index\;\%=\frac{\left(\text{W}2-\text{W}1\right)}{\text{W}1}\times 100$$

Where:

- W1 = Initial weight of the dry patch
- W2 = Weight of the swollen patch at a specific time point

#### 2.3.7 UV-Vis spectroscopy

UV-Vis spectra were acquired using an Agilent Cary 60 spectrophotometer (1 mm path length quartz cell) for both PWD and IBL containing patches. Each sample was scanned three times over 200–600 nm.

#### 2.3.8 *In vitro* drug release study

Drug release from the IBL patch was evaluated in phosphate-buffered saline (PBS, pH 7.4) and artificial saliva. After selecting the optimal PBS concentration (0.4x), patches were incubated in solution, and absorbance was measured at 10-minute intervals up to 60 minutes using a spectrophotometer (200–600 nm) (19).

#### 2.3.9 Fourier transform infrared spectroscopy and x-ray diffraction

FTIR spectra were recorded using a PerkinElmer UATR system to evaluate drug–excipient compatibility and identify characteristic functional groups. Samples were placed directly onto the ATR crystal, and spectra were collected over a wavenumber range of 4000–400 cm^-^¹. XRD analysis was performed using a Bruker D8 Advance system to determine the crystalline or amorphous state of the drug within the film. Samples included PWD and IBL patches, and individual polymers (EC, SALG, SCMC, HPMC). Diffraction patterns were recorded over a 2θ range of 10°–80° at a scanning rate of 2°/min. The degree of crystallinity was calculated from the crystalline intensity (I_200_) to that the intensity after subtracting amorphous peaks (I_200_-I_amorphous_) (20).


$$\%CI=\frac{I_{200}-I_{amorphous}}{I_{200}}\times 100$$

#### 2.3.10 Differential scanning calorimetry - thermogravimetric analysis

DSC was conducted (PerkinElmer DSC 8000) to investigate potential crystal rearrangements of Isotretinoin, Bromelain, and Limonene within the polymer matrix. Approximately 10 mg of each sample was scanned from 0 to 300 °C at 10 °C/min under nitrogen. TGA (PerkinElmer TGA 8000) assessed the thermal stability of the patch. Samples (10 mg) were heated from 30 to 850 °C at 10 °C/min under nitrogen.

#### 2.3.11 Filed emission scanning electron microscopy and energy-dispersive x-ray spectroscopy

Surface morphology and elemental compositions were examined using a ZEISS FESEM with EDX. Samples were mounted on brass stubs, coated with gold-palladium, and imaged under high vacuum.

#### 2.3.12 *In vitro* biocompatibility assay

Hemolysis assay was performed to evaluate the hemocompatibility of mucoadhesive patches according to ISO 10993–4 guidelines for blood-contacting biomaterials, assessing potential erythrocyte damage from patch leachables or surface interactions prior to *in vivo* application. Fresh human blood was collected from healthy volunteers following institutional ethical approval (KIDS/RES/66/2025 dated 24-11-2025) from the School of Dental Sciences, Kalinga Institute of Industrial Technology (Deemed to be University), Bhubaneswar, India. Samples were immediately transferred into K-EDTA coated tubes and maintained on ice to prevent coagulation. The blood was centrifuged at 1000 rpm for 10 min at 4 °C to isolate erythrocytes. The erythrocyte pellet was washed three times with phosphate-buffered saline (PBS, pH 7.4) until the supernatant became clear. The final pellet was resuspended in PBS to obtain a 2% (v/v) erythrocyte suspension for subsequent hemocompatibility analysis. Hemolytic potential was assessed by incubating patches with 0.9% NaCl and blood collected from healthy volunteers. Controls included distilled water (positive) and NaCl (negative). After 1 hour at 37 °C, samples were centrifuged, and the supernatant’s optical density at 545 nm was measured (21). Hemolysis was calculated as:


$$Hemolysis\;\%=\frac{\left(\text{OD of sample }-\text{ OD of negative control}\right)}{(\text{OD of positive control }-\text{ OD of negative control})\;}\times 100$$

#### 2.3.13 Ex vivo mucoadhesion time

Freshly excised goat buccal mucosa was used for *ex vivo* mucoadhesion studies. The tissue was mounted on a glass slide, and both IBL and PWD patches were wetted with phosphate buffer (pH 6.8), then gently pressed onto the mucosa for 30 seconds. The slide was placed in 200 mL of phosphate buffer (pH 6.8) at 37 °C with gentle stirring. The time until patch detachment was recorded as the mucoadhesion time (22).

### 2.4 Matrigel (type IV collagen) degradation assays

#### 2.4.1 Concentration and time dependent degradation

SDS-PAGE was performed to evaluate the degradation of Matrigel (Type IV collagen, 2 µg) in the presence of IBL patches containing bromelain. IBL patches with varying bromelain content (10–50 µg) were incubated with matrigel at 37 °C under defined conditions. Following incubation, samples were denatured and electrophoresed on 12% SDS-PAGE gels. Gels were stained with coomassie blue and analyzed using a gel documentation system. For time-dependent analysis, IBL patches containing 20 µg bromelain were incubated with Matrigel for 0–120 minutes. Control samples included matrigel incubated with patches lacking active components. Degradation was assessed based on changes in band intensity.

#### 2.4.2 Zymography assay for collagen degradation

Collagen degradation was further assessed using a zymography-based assay (Zymography Assay Kit, Abcam, Cat # ab234624) according to the manufacturer’s instructions. Type I collagen (2 µg) was incubated with IBL patches containing bromelain (equivalent to 20 µg), allowing enzymatic activity to be evaluated under assay conditions. Enzymatic activity reflects bromelain released from the IBL patch during the assay. A positive control was prepared using the enzyme standard provided with the kit, and all samples were processed under identical conditions. Fluorescence was measured at excitation/emission wavelengths of 490/520 nm using a photoluminescence spectrometer (FLS 1000, Edinburgh Instruments, UK). Enzymatic activity was calculated using a standard curve and expressed in pmol based on the following equation.


$$Enzyme\;activity\;(U/mL)=\frac{\text{B X }1000}{\text{A X C X D}}$$

where B is fluorescence intensity (RFU), A is the slope of the standard curve, C is sample volume (µL), and D is the dilution factor.

To assess the stability of bromelain embedded within the IBL patches, enzymatic activity was measured at defined storage intervals. Freshly prepared patches (0 h) served as the baseline reference, while stored patches were evaluated at subsequent time points (e.g., 3 and 6 months) under identical assay conditions. The percentage of retained enzymatic activity was calculated as:


$$Shelf\;life\;(\%\;Retained)=\frac{\text{Enzyme activity at time T}}{\text{Initial enzyme activity}}X\;100$$

### 2.5 *In vitro* biological evaluation

All *in vitro* experiments were performed using a uniform pharmaceutical-grade (pharma-grade) patch formulation across the different cell lines to ensure consistency and comparability.

#### 2.5.1 MTT (1-(4,5-dimethylthiazol-2-yl)-3,5-diphenylformazan) assay

SCC9 cells obtained from ATCC were culture in DMEM/F12 with 10% FBS and 1% Penstrep and McCoy, KB-3–1 cell lines (NCCS, Pune, India) were cultured in DMEM with 10% FBS and 1% Penstrep and. Cells (20,000/well) were seeded in 96-well plates and incubated for 24 hours. IBL patch and individual drugs, isotretinoin, bromelain and limonene were used at concentrations ranging from 2 to 1000 µg/mL. After 24 hours, MTT solution (0.5 mg/mL) was added, and cells were incubated for 4 hours. Formazan crystals were solubilized, and absorbance was measured at 570 nm. Based on the IC_50_ values obtained for the IBL patch using the MTT assay, the concentrations selected for treatment were 250, 500, and 750 µg/mL for all the three cell lines.

#### 2.5.2 Colony forming assay

Cells (500/well) were seeded in 6-well plates and allowed to form colonies for 6 days. After 24 hours of treatments, colonies were fixed, stained with crystal violet, imaged, and quantified using ImageJ.

#### 2.5.3 Wound healing assay

Cell migration was assessed by scratch assay. Cells (5 × 10^4^/well) were seeded in 6-well plates. At 60–70% confluency, a scratch was made, and cells were treated with IBL patch and PWD. Migration was monitored microscopically (Zeiss Primovert Inverted Microscope) at different time points (0, 12 and 24 hours), and wound closure was quantified using ImageJ.

#### 2.5.4 Apoptosis assay

SCC9, KB-3–1 and McCoy cells (2 × 10^4^/well) were seeded in 6-well plates and treated with IBL patch and PWD for 24 hours. After the incubation period, cells were fixed, stained with acridine orange/ethidium bromide (100 µg/mL each), and visualized under a fluorescence microscope to assess apoptosis.

#### 2.5.5 LPS induced acute inflammatory response in SCC9 cells and qRT-PCR analysis

SCC9 cells were cultured in DMEM supplemented with 10% FBS and antibiotics at 37 °C in a humidified 5% CO_2_ atmosphere. Cells were seeded at a density of 1 × 10^5^ cells per well in 6-well plates and allowed to adhere for 24 h. Experimental groups included untreated control, PWD-treated, IBL Patch-treated, LPS-induced (1 µg/mL), LPS + PWD and LPS + IBL-Patch. For LPS-induced groups, cells were stimulated with LPS for 24 h, later the cells were treated for an additional 24 h with PWD and IBL patch at IC_50_ concentrations. Non-LPS groups received the respective treatments directly without prior induction. This system was used to evaluate cytokine-associated inflammatory responses and does not represent a fibrosis model.

Assays were performed in triplicate. Following treatment, total RNA was extracted from cells using TRIzol reagent per the manufacturer’s protocol. Complementary DNA (cDNA) was synthesized from 1 µg RNA using the iScript cDNA Synthesis Kit (Cat# 1708891, Bio-Rad). Quantitative real-time PCR (qRT-PCR) was performed using iTaq Universal SYBR Green Supermix (Cat# 1725125, Bio-Rad), specific primers for IL-6, TNF-α, IL-2, TGF-β, and MMP-2 in 10 µL reactions on a QuantStudio system (Applied Biosystems). Cycling conditions consisted of 95 °C for 15 s (denaturation) followed by 40 cycles of 60 °C for 60 s (combined annealing/extension). Reactions were run in triplicate, with data normalized to GAPDH and relative mRNA expression calculated by the 2^−ΔΔCt method. Primer sequences are provided in **Supplementary Table 2**.

#### 2.5.6 Western blot analysis

Cells were lysed on ice in 250 µL RIPA buffer (20mM Tris, 1mM EDTA, 0.5mM EGTA, 0.1% Sodiumdeoxycholate, 150mM sodium chloride, 1% NP40, and 10% glycerol), containing protease inhibitor (Thermofisher Scientific, Cat # 1862209) for 30 min. Protein concentration was measured by Bradford assay KIT (Himedia, Cat # ML178-1PK, and 30 µg total protein was separated by 12% SDS-PAGE and transferred onto PVDF membranes (Merck Millipore, Cat#IPVH00010). Membranes were blocked using 5% (w/v) skimmed milk in TBST for 2 h at room temperature to prevent non-specific binding. Subsequently, the membranes were incubated overnight at 4 °C with primary antibodies diluted (1:1000) in 1× TBST. After thorough washing, membranes were exposed to the appropriate HRP-conjugated secondary antibodies, followed by signal detection according to the manufacturer’s protocol. Proteins were detected using Clarity Western ECL Substrate (Cat # 170-5060, BioRad) in the Bio-Rad ChemiDoc MP system, and expression levels were quantified relative to control using Image J software. The following antibodies were used: CK17, CK18, COX2 and GAPDH (Elabsciences).

#### 2.5.7 Immunocytochemistry

Cells cultured on coverslips were washed with PBS and fixed in 4% paraformaldehyde (Santa Cruz, Cat # 30525-89-4) for 20 min at room temperature (RT). Following fixation, cells were permeabilized with 0.2% Triton X-100 (Sigma, Cat # 9036-19-5) for 5 min on ice and washed three times with PBS (10 min each). Cells were blocked with 1% BSA (Himedia, Cat # MB083) for 30 min at RT, then incubated with primary antibodies against CK17 and COX2 (Elabscience; 1:300 dilution) for 1 h at RT, followed by three PBS washes (10 min each). Secondary goat anti-rabbit IgG antibody incubation was performed, with subsequent PBS washing (3 × 10 min). Finally, cells were mounted inverted using ProLong™ Gold Antifade Mountant with DAPI (Thermo Fisher Scientific, Cat # P36931) and imaged by fluorescence microscopy and the fluorescence intensity was quantified relative to control using Image J Software.

### 2.6 Histological analysis in rat tail

Histopathological evaluation of rat tail tendon tissue was performed to assess potential structural alterations following *ex vivo* exposure to the mucoadhesive buccal patch. Tendon tissues were obtained from discarded specimens derived from unrelated, institutionally approved animal studies. No animals were euthanized specifically for the present investigation. All procedures complied with the guidelines of the Committee for the Purpose of Control and Supervision of Experiments on Animals (CPCSEA), Government of India, and were approved by the Institutional Animal Ethics Committee (IAEC) of Sri Ramachandra Institute of Higher Education and Research, Chennai, India (Approval No. IAEC/74/SRIHER/924/2024).

Rat tail tendon tissues were incubated with PWD and IBL patches at 37 °C for 2 h. Following incubation, tissues were fixed overnight in 10% neutral buffered formalin. Samples were dehydrated through a graded ethanol series (60%, 70%, 80%, 96%, and 100%), cleared, and embedded in paraffin wax. Paraffin blocks were sectioned at 4–5 µm using a rotary microtome, and sections were mounted on glass slides and prewarmed at 70 °C for 15 min. Sections were deparaffinized using three changes of xylene (15 min each), followed by rehydration through graded ethanol (three changes of 100% ethanol, 5 min each) and rinsing under running tap water for 5 min prior to staining.

#### 2.6.1 Hematoxylin and eosin staining

Paraffin-embedded sections of rat tail tendon and rabbit buccal mucosa were subjected to Hematoxylin and Eosin (H&E) staining for general histological evaluation. Sections were stained with hematoxylin for 10 min, rinsed in running tap water (2 min), differentiated in 1% acid alcohol, and blued in saturated lithium carbonate solution. After washing, sections were counterstained with eosin for 1 min. Dehydration was performed using 80% ethanol (2 min, twice) followed by 100% ethanol (30 s, twice), cleared in xylene, and mounted with DPX mountant. Slides were examined under a light microscope for morphological assessment.

#### 2.6.2 Picrosirius red staining

Picrosirius Red (PSR) staining was performed to assess collagen deposition and fiber organization using a commercially available kit (Abcam, Cambridge, UK; Cat. No. AB150681) according to the manufacturer’s protocol. Deparaffinized sections were rehydrated through graded ethanol, rinsed in distilled water, and stained with Picrosirius Red solution for 1 h at room temperature. Sections were briefly differentiated in 70% ethanol followed by absolute ethanol, cleared in xylene, mounted with DPX, and examined under polarized light microscopy to evaluate collagen fiber birefringence and distribution.

#### 2.6.3 Masson trichome staining

Masson’s Trichrome (MT) staining was performed to evaluate collagen deposition and fibrosis using a commercial kit (Abcam, Cambridge, UK; Cat. No. 1.00485.0001) according to the manufacturer’s instructions. Sections were stained with Weigert’s iron hematoxylin, followed by sequential treatment with Azophloxine, Tungsto-phosphoric acid–Orange G, and Light Green solutions. After dehydration through graded ethanol, sections were cleared in xylene, mounted with DPX, and examined under light microscopy to assess collagen distribution and tissue architecture.

### 2.7 *In vivo* study

*In vivo* evaluation was performed using a carbon tetrachloride (CCl_4_) induced inflammation model to assess the effects of the formulation under fibrotic conditions. A total of 21 male New Zealand white rabbits were randomly allocated into three groups: G1 (normal control, n = 3), G2 (patch without drug, PWD, n = 9), and G3 (test patch containing isotretinoin, bromelain, and limonene, n = 9). Group G1 received no induction or treatment. Groups G2 and G3 were each subdivided into three treatment-duration subgroups: 2 weeks (A), 4 weeks (B), and 6 weeks (C), with three animals per subgroup (**Supplementary Table 3**).

Oral fibrotic model was induced in G2 and G3 using CCl_4_ (4% solution, 0.4 mL per dose) administered into the buccal submucosa on alternate days for 78 days under local anesthesia (10% lidocaine spray). Animals were monitored throughout the induction phase for body weight, clinical signs, and oral mucosal changes. Following induction, treatment commenced on day 82. Subgroups G2A, G2B, and G2C received PWD, while G3A, G3B, and G3C received the IBL mucoadhesive buccal patch, applied twice weekly for 2, 4, or 6 weeks according to subgroup allocation. The experimental timeline consisted of an induction phase (up to 9 weeks) followed by a treatment phase beginning on day 82. Measurement weeks (Weeks 3, 6, and 9 during induction; Weeks 10, 13, and 16 during treatment) represent cumulative weeks post-randomization. This clarification is consistently reflected in all tables and figure legends.

For patch application, animals were anesthetized using ketamine (25 mg/kg) and xylazine (3 mg/kg) administered via the intramuscular route prior to patch application. The patch was positioned in direct contact with the fibrotic buccal mucosa and maintained for 2 hours, during which food and water were withheld. Animals were continuously monitored during the treatment phase for oral mucosal changes, mouth opening (measured using a Vernier caliper), body weight, and general health status. At the end of each subgroup’s treatment period, animals were euthanized under intramuscular anesthesia with ketamine (25 mg/kg body weight) and xylazine (3 mg/kg body weight), followed by an intravenous overdose of thiopentone sodium (90 mg/kg). Rabbits that are found dead during the study period were subjected to post-mortem examination. Buccal tissues were collected for histological evaluation, including assessment of epithelial integrity, collagen deposition, and tissue architecture. All animal procedures were conducted in accordance with institutional ethical guidelines and approved by the relevant ethics committee.

### 2.8 Statistical analyses

Data are presented as mean ± standard deviation (SD). Statistical analysis was performed using GraphPad Prism (version 8.0, USA). Comparisons between groups were conducted using one-way ANOVA followed by appropriate *post hoc* tests. A p-value < 0.05 was considered statistically significant.

## 3 Results and discussion

### 3.1 Characterization of mucoadhesive buccal IBL patch

#### 3.1.1 Physical characterization of IBL patch

The IBL loaded MABPs appeared translucent with a smooth and flexible surface, and no visible drug aggregation within the matrix. All formulations exhibited uniform thickness and weight, with thickness ranging from 3.2 ± 0.1 to 3.93 ± 0.05 mm and weight (1 × 1 cm²) between 127.1 ± 1.1 and 167.86 ± 1.55 mg (**Table 1**). The minimal variation across batches indicates reproducibility of the casting process (24). pH values ranged from 6.31 ± 0.05 to 6.43 ± 0.05, within the physiological salivary range (6.3–7.3) (25). The optimized formulation (F7) exhibited a pH of 6.31 ± 0.05, suggesting compatibility with buccal mucosa.

Mechanical performance varied notably across the formulations, as reflected by their folding endurance. F7 achieved the greatest resistance to repeated folding (257 ± 1.53 folds), whereas PWD showed a lower but still appreciable level of flexibility (182 ± 1.50 folds). The reduction after drug incorporation may be attributed to structural changes within the polymer matrix; however, all values remained within an acceptable range for MABPs (26). Drug content uniformity ranged from 95.3 ± 0.87% to 98.96 ± 0.49%, indicating consistent distribution of active components (**Supplementary Figure 1**) (30).

UV–Vis spectrophotometric analysis confirmed absorbance peaks corresponding to limonene (232 nm), bromelain (283 nm), and isotretinoin (346 nm). Spectra obtained in PBS (pH 7.4) and artificial saliva demonstrated consistent drug detection (**Figure 1b**). This method is widely applied in pharmaceutical analysis for quantitative assessment of drug content and release behavior (25, 27–29). To evaluate release under different pH conditions, the IBL patch was assessed in PBS at pH 4, 7, and 10. Drug release was observed at all pH levels, with variations in release sequence among components (**Supplementary Figure 2**). Increased drug concentration corresponded to increased absorbance values, reflecting proportional drug release. These findings indicate stability of release behavior under varying conditions relevant to the oral environment.

Based on physicochemical parameters including thickness, weight variation, surface pH, folding endurance, and drug content uniformity, eight formulations (F1–F8) were compared. Among these, F7 demonstrated balanced characteristics and was selected for further studies. Formulation F7 (SALG: SCMC: HPMC, 1%:2.5%:1.5%) with 40 mg/patch loading was selected based on balanced physicochemical and functional parameters. F7 exhibited thickness of 3.46 ± 0.08 mm, weight of 127.1 ± 1.1 mg, surface pH of 6.31 ± 0.05, folding endurance of 257 ± 1.53 folds, and drug content uniformity of 98.36 ± 0.92%. The polymer composition (SALG, SCMC, and HPMC) influenced hydration and film integrity. Variations in polymer ratios affected flexibility and swelling behavior, consistent with previous reports on mucoadhesive systems (31, 32). Although isotretinoin is poorly water-soluble, its low loading (0.05% w/w) and incorporation within hydrophilic polymers (HPMC, SCMC, SALG) likely facilitated dispersion within the matrix. Hydrophilic polymer systems are commonly used to stabilize lipophilic compounds in amorphous or dispersed states (33, 34). Swelling studies showed that the IBL patch exhibited a mean swelling index of 18.27% at 8 h compared to 12.57% for the patch without drug (**Supplementary Table 4**). Hydrophilic polymer components contributed to water uptake and matrix expansion, influencing drug diffusion behavior (35).

*In vitro* release studies demonstrated cumulative release in PBS of 86.97% (limonene), 86.6% (bromelain), and 87.1% (isotretinoin) within 60 min. In artificial saliva, release values reached 89.75%, 88.07%, and 97.8%, respectively (**Figures 1E, F**). The release profile showed an initial increase followed by gradual progression over time, consistent with reported mucoadhesive buccal systems (36). Release kinetics were influenced by polymer swelling and gel formation, particularly by SCMC and HPMC content (37, 38). Polymer viscosity and chain entanglement can modulate diffusion rates, thereby affecting drug release behavior (39, 40). Collectively, these findings indicate that formulation F7 exhibited reproducible physicochemical characteristics, controlled swelling behavior, and consistent release profiles, supporting its selection for subsequent *in vitro* and *in vivo* evaluation.

#### 3.1.2 FTIR and XRD analysis

**Figure 2:**
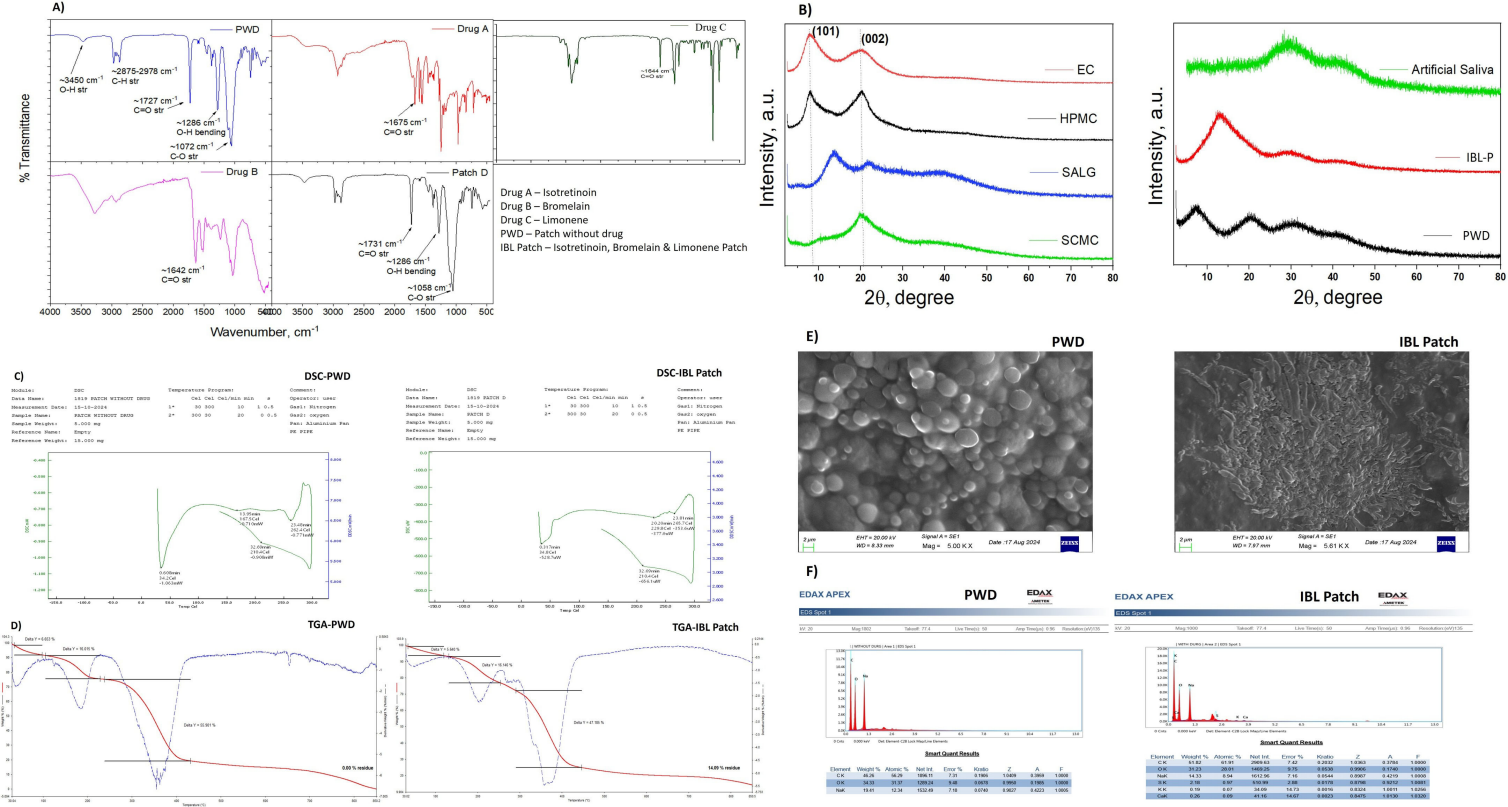
Physicochemical characterization of the IBL patch. **(A)** FTIR spectra of PWD, isotretinoin (Drug A), bromelain (Drug B), limonene (Drug C), and the drug-loaded IBL patch (IBL-P). **(B)** XRD patterns of EC, HPMC, SALG, SCMC, PWD, IBL-P, and IBL-P after exposure to artificial saliva (AS). **(C)** DSC thermograms of PWD and IBL-P. **(D)** TGA thermograms showing thermal degradation profiles of PWD and IBL-P. **(E)** SEM micrographs illustrating surface morphology of PWD and IBL-P. **(F)** EDX spectra confirming elemental composition of PWD and IBL-P.

The FTIR spectrum of the patch without drug displayed a broad absorption band around 3450 cm^-^¹, attributed to O–H stretching vibrations, and a corresponding O–H bending peak at 1286 cm^-^¹, confirming the presence of hydroxyl groups within the polymer matrix. Distinct peaks at 1642, 1644, and 1675 cm^-^¹ were observed for the drug molecules, corresponding to C=O stretching vibrations of the carboxylate groups in isotretinoin, bromelain, and limonene, respectively. The polymer mixture exhibited a characteristic C–O stretching band at 1072 cm^-^¹, which shifted to 1058 cm^-^¹ upon drug incorporation. This shift suggests the formation of hydrogen bonds between the drugs and the polymer’s –OH groups, indicating successful molecular interaction and integration of the active compounds within the patch (**Figure 2A**). These spectral features are consistent with previous reports for isotretinoin, limonene, and bromelain (41, 42), further validating the present formulation.

XRD patterns revealed that the base polymers (EC, HPMC, SALG, SCMC) were semi-crystalline in nature, with two broad peaks at 2θ = 8.1° (d = 10.9 Å, (101) plane) and 2θ = 20.3° (d = 4.4 Å, (002) plane), reflecting interchain distances (41, 42)) (**Figure 2B**). The calculated degree of crystallinity (CI) was 41.9% for EC, 40.46% for HPMC, 27.45% for SALG, and 8.4% for SCMC (**Figure 2B**). Upon drug loading, the crystallinity of the polymer matrix decreased, as evidenced by the broadening and reduction in peak intensity. This reduction in crystallinity is attributed to the disruption of hydrogen bonding and increased chain flexibility, resulting in the formation of amorphous regions. Such structural changes enhance patch hydrophilicity, reduce brittleness, and facilitate improved drug release, particularly in artificial saliva (43–45).

#### 3.1.3 DSC-TGA analysis

DSC analysis revealed a distinct exothermic melting peak at 262.4 °C for the PWD, indicative of its crystalline nature. The optimized IBL patch exhibited a slightly altered thermogram, with broadening and shifting of the melting peak, consistent with the dispersion of drugs in an amorphous form within the polymer matrix. The presence of 14.09% residue at 850 °C further supports successful drug incorporation (**Figure 2C**). These results indicate good compatibility among the patch constituents and are consistent with previous findings, as also supported by FTIR analysis https://www.formulationbio.com/oral-thin-film/differential-scanning-calorimetry-dsc-analysis-for-oral-thin-film.html (46).

TGA analysis showed a three-stage weight loss profile for both patches. The initial 5–16% weight loss (30–200 °C) was attributed to moisture evaporation, while the main decomposition occurred at 325–355 °C for the PWD (55.9% loss) and at 340 °C for the IBL patch. Notably, the IBL patch retained 14.09% residue at 850 °C, reflecting the thermal stability imparted by the drug components (**Figure 2D**). The increased residual mass and altered decomposition profile after drug loading confirm enhanced thermal stability and successful formulation modification (47).

#### 3.1.4 SEM and EDX

SEM imaging of the PWD revealed a smooth, continuous surface, free from pores or agglomerates, indicative of uniform film formation. After loading with isotretinoin, bromelain, and limonene, the IBL patch exhibited a more inhomogeneous, flake-like surface morphology, reflecting increased porosity and microstructural changes due to drug incorporation (**Figure 2E**). This morphological transformation is known to enhance mucoadhesive and drug delivery properties (48). EDX analysis confirmed the elemental composition of both the control and IBL patches. Patch without drug primarily contained carbon (38.84–47.13 wt%), oxygen (33.66–47.25 wt%), and sodium (13.91–20.46 wt%), consistent with the polymer matrix. The IBL patch showed similar major elements, with the addition of sulfur (up to 2.18 wt%), potassium, calcium, and chlorine in trace amounts, confirming successful drug incorporation and homogeneity (**Figure 2F**). These results collectively support the patch’s suitability for mucoadhesive and buccal drug delivery applications.

#### 3.1.5 Hemolysis assay

Hemocompatibility was evaluated by measuring the hemolytic potential of both the IBL and PWD patches. Both formulations exhibited negligible hemolysis over a 2-hour incubation period, with hemolysis percentages comparable to the negative control (**Supplementary Figure 3**). These findings confirm the non-toxic nature of the polymeric matrices toward erythrocytes and support their suitability for *in vivo* applications which is preliminary observation. The results are consistent with previous studies (49, 50) that reported minimal hemolytic activity for similar drug-loaded polymer patches, even after extended exposure.

#### 3.1.6 Ex vivo mucoadhesive time

The *ex vivo* mucoadhesive time, assessed using goat buccal mucosa, was significantly extended for the IBL patch (3.05 ± 0.308 hours) compared to the PWD (1.34 ± 0.04 hours). This enhanced mucoadhesion is attributed to the optimized polymer composition, particularly the inclusion of hydrophilic polymers such as HPMC, which increase both swelling capacity and adhesion strength. The resulting hydrated polymeric gel layer enhances mucoadhesion and prolongs the patch’s residence time on the mucosal surface, consistent with previous reports. The correlation between higher polymer viscosity and increased mucoadhesive time highlights the critical role of polymer selection and formulation in achieving prolonged mucosal contact (51).

### 3.2 Degradation of human type IV collagen by IBL patch

**Figure 3:**
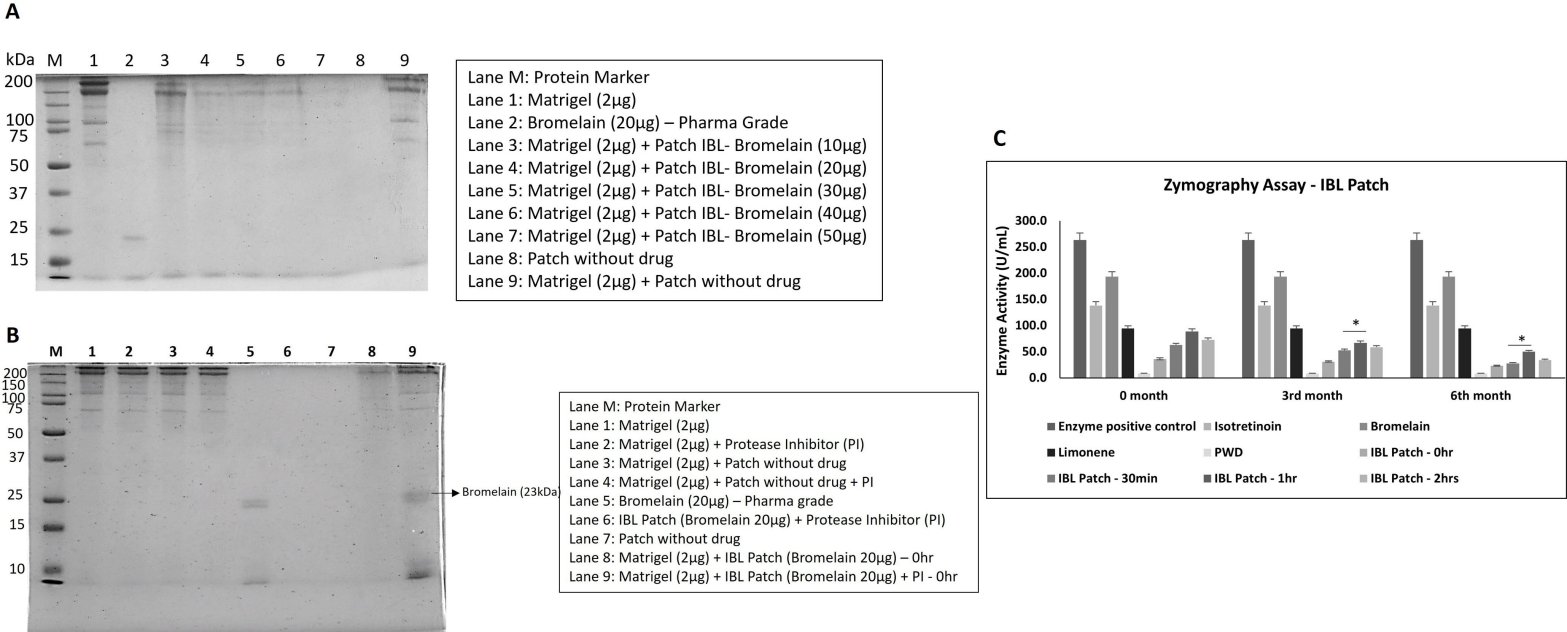
Degradation of collagen by bromelain loaded in IBL patch. SDS-PAGE analysis of **(A)** concentration dependent and **(B)** Inhibition of Matrigel degradation by the IBL patch in the presence of a protease inhibitor, confirming the enzymatic specificity of bromelain embedded in IBL patch. **(C)** Zymography assay showing enzymatic activity of IBL patches at 0, 3, and 6 months. Enzyme positive control (collagenase) and PWD were included as references. Data are presented as mean ± SEM, n=3. *p < 0.05 compared to 0^th^ month.

SDS-PAGE analysis showed concentration- and time-dependent degradation of human type IV collagen following incubation with the bromelain containing IBL patch. Increasing bromelain-equivalent concentration (up to 50 µg/mL) was associated with a progressive reduction in the intensity of the characteristic α-1 and α-2 collagen bands (**Figure 3A**). Time-course analysis indicated partial degradation within 1 h and a marked reduction in detectable collagen bands after 2 h of incubation (**Supplementary Figure 4**), suggesting retention of proteolytic activity under the experimental conditions.

Inhibition studies using a protease inhibitor reduced collagen degradation (**Figure 3B**), indicating that the observed matrix breakdown was enzyme-dependent. The reduction in α-chain band intensity with increasing concentration and incubation time is consistent with established approaches for assessing collagenolytic activity (23). Similar collagen degradation patterns have been reported for bromelain and related proteolytic enzymes in extracellular matrix models (49, 52). Collagen degradation observed in this study is attributable to the proteolytic activity of bromelain released from the IBL patch, as confirmed by concentration, time, and inhibitor dependent assays.

Zymography assays were performed to quantify enzymatic activity over time and during storage. At 1 h incubation, activity decreased from 88.90 U/mL (0 month) to 66.83 U/mL (3 months) and 50.2 U/mL (6 months). At 2 h incubation, activity declined from 70.49 U/mL (0 month) to 54.63 U/mL (3 months) and 33.9 U/mL (6 months). These findings indicate a gradual reduction in enzymatic activity during storage when incorporated within the polymer matrix. Control experiments supported assay specificity. Collagenase (positive control) showed consistently high activity (263.5 U/mL), while the PWD exhibited minimal activity (8.35 U/mL). Individual components, including bromelain, isotretinoin, and limonene, showed measurable activity under assay conditions (**Figure 3C**).

Shelf-life analysis indicated that the IBL patch retained 75.2% and 56.5% enzymatic activity at 3 and 6 months, respectively, at 1 h incubation, and 80.9% and 46.8% at 2 h incubation. Although activity decreased over time, measurable proteolytic function was maintained during the storage period. The enzymatic activity observed for the IBL patch reflects retained functional activity within the formulation matrix and is not directly comparable to purified collagenase used as a positive control. The observed collagen degradation profile is consistent with previous reports of bromelain mediated hydrolysis of collagen in fish collagen, rat tail, and rabbit models (23, 42). These results support the functional retention of bromelain activity within the IBL patch under experimental conditions.

### 3.3 *In vitro* cytotoxicity and functional assays

The *in vitro* cytotoxic and functional evaluation of the IBL mucoadhesive patch showed concentration dependent effects in SCC9 and KB-3–1 oral squamous carcinoma cells, while comparatively limited effects were observed in normal fibroblast (McCoy) cells under the tested conditions.

**Figure 4:**
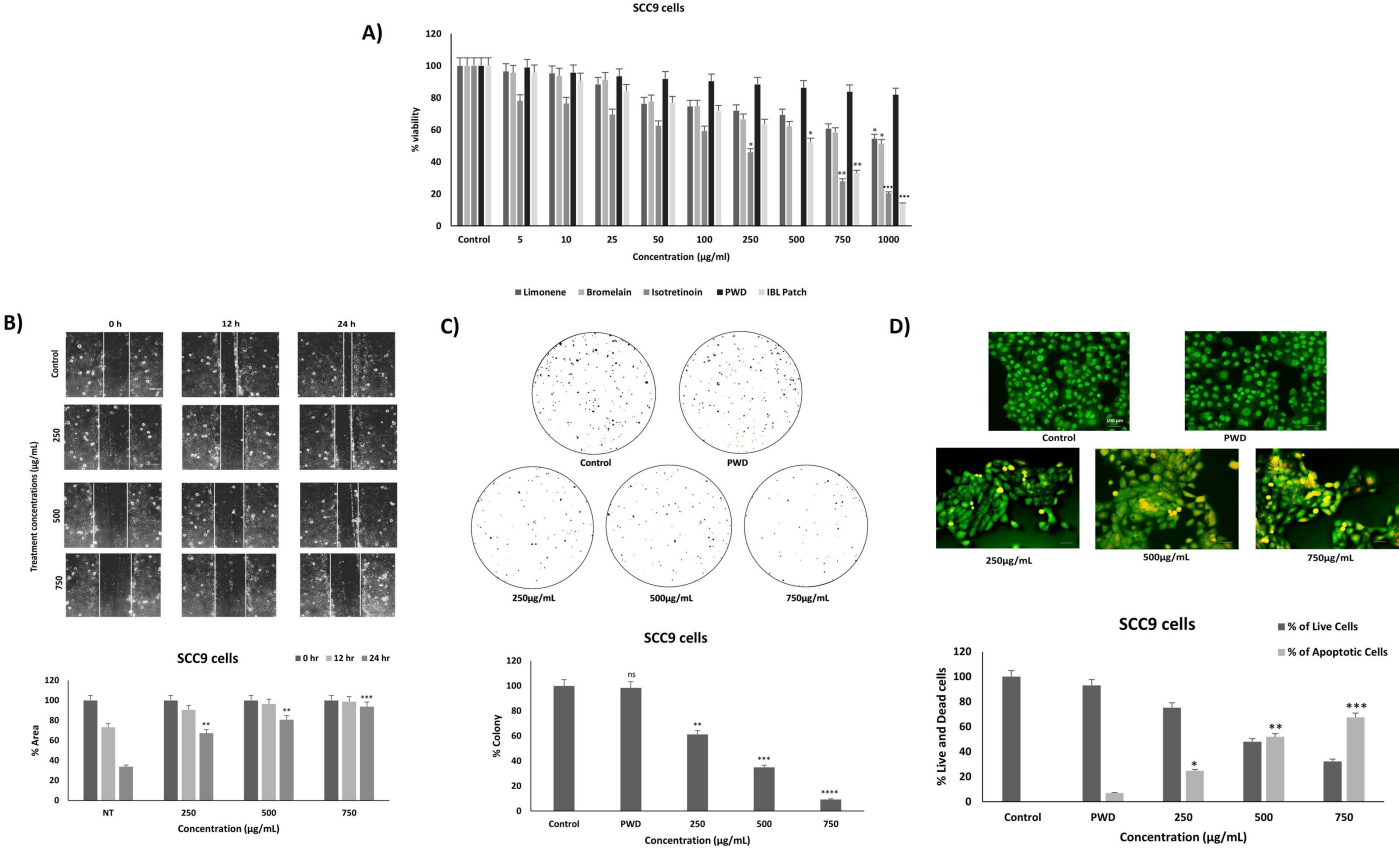
*In vitro* cytotoxic and functional effects of the IBL patch in SCC9 oral cancer cells. **(A)** MTT assay showing dose-dependent reduction in SCC9 cell viability following treatment with increasing concentrations of the IBL patch and individual bioactives. **(B)** Colony formation assay demonstrating decreased clonogenic survival in treated SCC9 cells. **(C)** Wound-healing assay illustrating impaired migratory capacity of SCC9 cells at 0, 12, and 24 h following treatment. **(D)** AO/EtBr dual staining indicating dose-dependent induction of apoptosis in SCC9 cells, with representative fluorescence images and quantitative analysis of live and apoptotic populations. Data are presented as mean ± SD (n = 3). Statistical significance was determined relative to untreated controls (*P < 0.05, **P < 0.01, ***P < 0.001 vs Control).

In MTT assays, isotretinoin and bromelain showed concentration-dependent reductions in oral cancer cell viability, whereas limonene maintained comparatively higher viability across tested concentrations. The IBL patch induced a dose-dependent decrease in SCC9 cell viability (**Figure 4A**), with an IC_50_ of approximately 487 µg/mL after 24 h exposure. Based on this response profile, subsequent functional assays were performed at 250, 500, and 750 µg/mL. Comparable concentration-dependent responses were observed in KB-3–1 cells (**Supplementary Figure 5A**), with a progressive reduction in viability across increasing concentrations and a calculated IC_50_ of approximately 530 µg/mL after 24 h exposure. In contrast, McCoy cells retained high viability across the tested concentration range (**Supplementary Figure 5B**). PWD did not show appreciable effects on cell viability. The observed response patterns were comparable across both cell lines under the tested conditions.

Clonogenic assays indicated a concentration-dependent reduction in colony-forming capacity in SCC9 cells with increasing concentrations of the IBL patch (**Figure 4B**), particularly at 500 and 750 µg/mL. A similar trend was observed in KB-3–1 cells (**Supplementary Figure 5C**), with reduced colony number and density at higher concentrations. In contrast, McCoy cells maintained relatively higher colony-forming efficiency across treatment groups (**Supplementary Figure 5D**). Wound-healing assays demonstrated delayed closure in SCC9 cells following treatment (**Figure 4C**), particularly at concentrations ≥500 µg/mL. A comparable reduction in migratory capacity was observed in KB-3–1 cells (**Supplementary Figure 5E**). In contrast, migration of McCoy fibroblasts remained largely comparable to untreated controls (**Supplementary Figure 5F**) under the tested conditions. AO/EtBr dual staining indicated concentration-dependent apoptotic morphology in SCC9 cells (**Figure 4D**). Similar morphological changes were observed in KB-3–1 cells (**Supplementary Figure 5G**). In contrast, McCoy cells exhibited minimal alterations under the same conditions (**Supplementary Figure 5H**). PWD did not induce appreciable apoptotic features.

The observed cellular responses are consistent with previously reported biological effects of isotretinoin (53, 54), bromelain (14), and limonene (9, 10). Reduced clonogenic survival and migration have also been described for bromelain and limonene in cancer models (55, 56), and apoptotic changes are consistent with earlier reports on these agents (57–59). Together, these findings confirm that incorporation of the bioactive components within the mucoadhesive matrix preserves biological activity while maintaining minimal effects on normal fibroblast cells under the tested conditions.

### 3.4 Gene expression profiles

**Figure 5:**
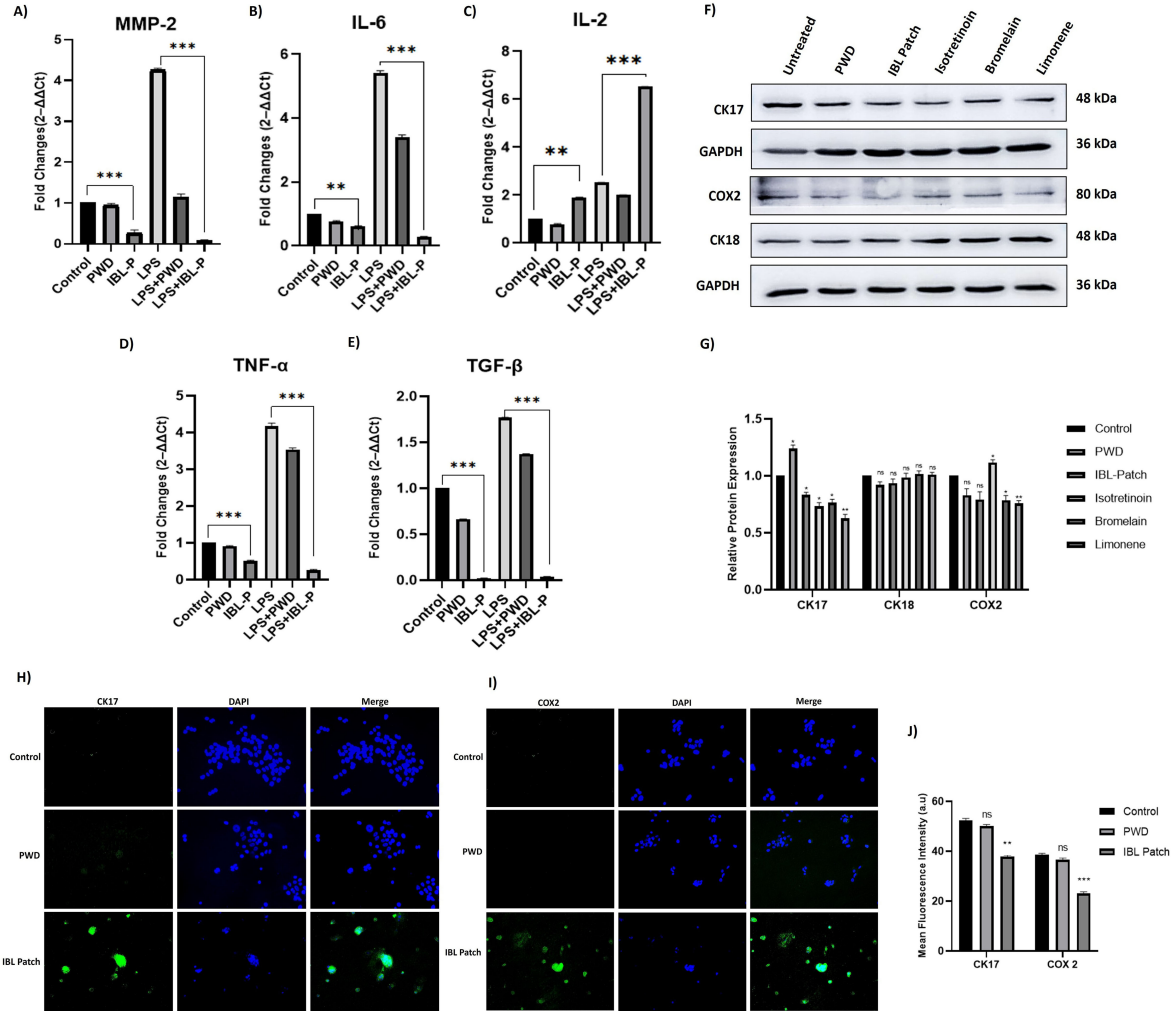
IBL patch attenuates inflammatory responses and modulates epithelial markers in SCC9 cells. **(A–E)** Relative mRNA expression of MMP-2, IL-6, IL-2, TNF-α, and TGF-β under indicated treatment conditions, analyzed by qRT-PCR and normalized to an internal control (2^–ΔΔCt method). Data are presented as mean ± SD (n = 3). **(F)** Representative Western blots of CK17, CK18, and COX-2 protein expression; GAPDH served as loading control (n = 3). **(G)** Densitometric quantification of protein levels normalized to GAPDH (mean ± SD, n = 3). **(H–J)** Immunofluorescence images showing CK17 and COX-2 (green) with DAPI nuclear staining (blue), and corresponding fluorescence intensity quantification under Control, PWD, and IBL-Patch treatments. Statistical analysis was performed using one-way ANOVA followed by Tukey’s *post hoc* test. *p < 0.05, **p < 0.01, ***p < 0.001; ns, not significant.

Quantitative real-time PCR analysis revealed differential regulation of inflammatory and extracellular matrix (ECM)-associated genes in SCC9 cells under various treatment conditions (**Figures 5A–E**). Relative expression levels were calculated using the 2^−ΔΔCt method and normalized to GAPDH. LPS stimulation increased expression of MMP-2 (4.2-fold), IL-6 (5.6-fold), and TNF-α (4.1-fold) compared to control, indicating activation of inflammatory and ECM remodeling pathways. Co-treatment with the IBL patch was associated with reduced expression of MMP-2 (1.3-fold), IL-6 (3.3-fold), and TNF-α (3.5-fold) relative to LPS-treated conditions (p < 0.001). PWD and IBL patch alone showed minimal changes (0.4–1.2-fold).

IL-2 expression showed a moderate increase following LPS (2.5-fold), with a further increase observed in the LPS + IBL patch group (6.4-fold, p < 0.001 vs. LPS). TGF-β expression was modestly elevated with LPS (1.7-fold), while the LPS + IBL patch group showed reduced expression (0.5-fold vs. control, p < 0.001). The observed changes indicate alterations in cytokine-associated inflammatory responses and ECM related responses under the tested conditions. However, the functional interpretation of IL-2 and TGF-β expression is complex, as their roles are context-dependent and may vary across different stages of inflammation and fibrosis. Therefore, the changes in IL-2 and TGF-β expression are presented descriptively without assigning specific functional implications. The underlying mechanisms and cellular sources contributing to these expression changes require further investigation (61, 62). These observations are consistent with cytokine expression patterns reported in OSCC-related inflammatory models and may provide contextual relevance to inflammatory processes associated with OSMF (63, 64).

### 3.5 Protein expression profiles

Western blot analysis (three independent biological replicates) demonstrated regulation of selected epithelial and inflammatory markers in SCC9 cells following treatment (**Figures 5F, G**). Densitometric values were normalized to GAPDH. A reduction in CK17 expression was observed following IBL patch treatment compared to untreated control. Individual components (isotretinoin, bromelain, and limonene) produced comparatively modest changes. COX-2 expression was reduced in the IBL-treated group relative to control, whereas individual components resulted in partial reductions. CK18 expression remained stable across all experimental conditions. Consistent GAPDH expression confirmed equal protein loading. The observed reduction in COX-2 expression aligns with decreased prostaglandin-mediated inflammatory signaling downstream of cytokine activation. Similarly, modulation of CK17 expression suggests restoration of epithelial homeostasis disrupted during chronic inflammation (65–67). The stability of CK18 suggests preservation of epithelial structural characteristics under the tested conditions.

### 3.6 Fluorescence imaging profiles

Immunofluorescence analysis supported the protein-level observations (**Figures 5H, I**). CK17 staining demonstrated cytoplasmic expression in control and PWD-treated cells, while reduced fluorescence intensity was observed following IBL patch treatment. COX-2 showed constitutive cytoplasmic fluorescence in control and PWD conditions, which was attenuated in the IBL-treated group. DAPI counterstaining indicated comparable cell density across groups.

Quantitative ImageJ analysis (n = 3 biological replicates; three random fields per condition; scale bar: 100 μm; DAPI-normalized) demonstrated significant reductions in mean fluorescence intensity (MFI) following IBL patch treatment. CK17 MFI decreased from 52.3 ± 0.8 (Control) and 50.1 ± 0.6 (PWD) to 37.9 ± 0.5 (↓27.5% vs. Control, p < 0.01). COX-2 MFI decreased from 38.6 ± 0.6 (Control) and 36.6 ± 0.7 (PWD) to 23.1 ± 0.6 (↓40.2% vs. Control, p < 0.001). PWD remained statistically comparable to Control (p > 0.05) (**Figure 5J**). These findings are consistent with western blot and qPCR data, indicating coordinated modulation of inflammatory and epithelial markers under the tested conditions (60). CK17 and COX-2 are established markers associated with epithelial dysregulation and inflammatory signaling in OSCC/OSMF contexts (65–67).

### 3.7 Ex vivo ECM remodeling study

Hematoxylin and Eosin (H&E) staining revealed differences in epithelial morphology between control and treated specimens. Control tissues exhibited a thicker epithelium (6–8 cell layers) with an intact superficial keratin layer, whereas treated specimens showed a comparatively thinner epithelium (4–5 cell layers) with partial keratin detachment (**Supplementary Figures 6A, B**). The underlying connective tissue appeared densely packed in both groups; however, H&E staining did not permit detailed evaluation of collagen organization. Picrosirius Red staining under polarized light provided enhanced assessment of collagen architecture. Control tissues demonstrated strong orange-red birefringence, consistent with well-organized collagen bundles. In contrast, treated tissues exhibited thinner and less organized fibers with reduced polarization intensity and mixed orange-red, yellow, and greenish-yellow birefringence (**Supplementary Figures 6C, D**). These changes suggest alterations in collagen fiber thickness and organization. In addition, semi-quantitative analysis of PSR birefringence demonstrated a relative reduction in orange/red (thick/mature) collagen fibers and a corresponding increase in green/yellow (thin/immature) fibers in treated specimens compared to controls, along with a decrease in total collagen area (**Supplementary Figure 6G**).

Masson–Goldner Trichrome (MT) staining further supported these findings. Control specimens showed intense and uniform green staining corresponding to dense collagen bundles beneath the epithelium (**Supplementary Figure 6F**). Treated tissues demonstrated comparatively reduced and heterogeneous green staining, with looser bundle arrangement and increased inter-fibrillar spacing (**Supplementary Figure 6E**). Nuclear and cytoplasmic morphology remained preserved in both groups, indicating maintenance of overall tissue architecture. The inability of H&E staining to differentiate collagen subtypes highlights the value of PSR staining for connective tissue characterization (68, 69). Under polarized microscopy, orange-red birefringence has been associated with thicker collagen fibers, whereas yellow and greenish-yellow patterns are often reported in thinner or less densely organized fibers (70, 71). Reduced polarization intensity and altered fiber distribution in treated specimens are consistent with modifications in ECM organization (72, 73). The utility of PSR in detecting such structural differences has been widely documented (74, 75). Similarly, MT staining demonstrated qualitative differences in collagen density and distribution without evidence significant tissue damage (76). When interpreted together, PSR and MT findings indicate partial structural reorganization of collagen fibers under *ex vivo* conditions (77). However, as these staining techniques are qualitative to semi-quantitative, the results should be interpreted as histological evidence of ECM interaction rather than definitive antifibrotic efficacy.

### 3.8 *In vivo* chemically induced experimental fibrosis model in rabbit

Oral potentially malignant disorders (OPMDs) are characterized by mucosal alterations associated with an increased risk of malignant transformation. In this study, the performance of the mucoadhesive IBL patch was evaluated in a chemically induced rabbit. Inflammatory lesions were induced in New Zealand white rabbits through alternate-day buccal submucosal injections of 0.4 mL of 4% carbon tetrachloride (CCl_4_) over 78 days. CCl_4_ was selected based on its established fibrogenic properties in experimental fibrosis models (78, 79). Animals were allocated into normal controls (G1), patch without drug groups (PWD; G2A–C), and IBL-treated groups (G3A–C), corresponding to 2, 4, and 6 week treatment durations. Patches were applied twice weekly under anesthesia and maintained in contact with the buccal mucosa for 2 h. No mortality or signs of systemic toxicity was observed during the induction or treatment phases. Induced animals developed pale to whitish buccal mucosa with increased firmness on palpation, consistent with fibrotic changes. Mouth opening was significantly reduced in all induced groups compared to normal controls (p < 0.05–0.01), indicating functional restriction.

**Figure 6:**
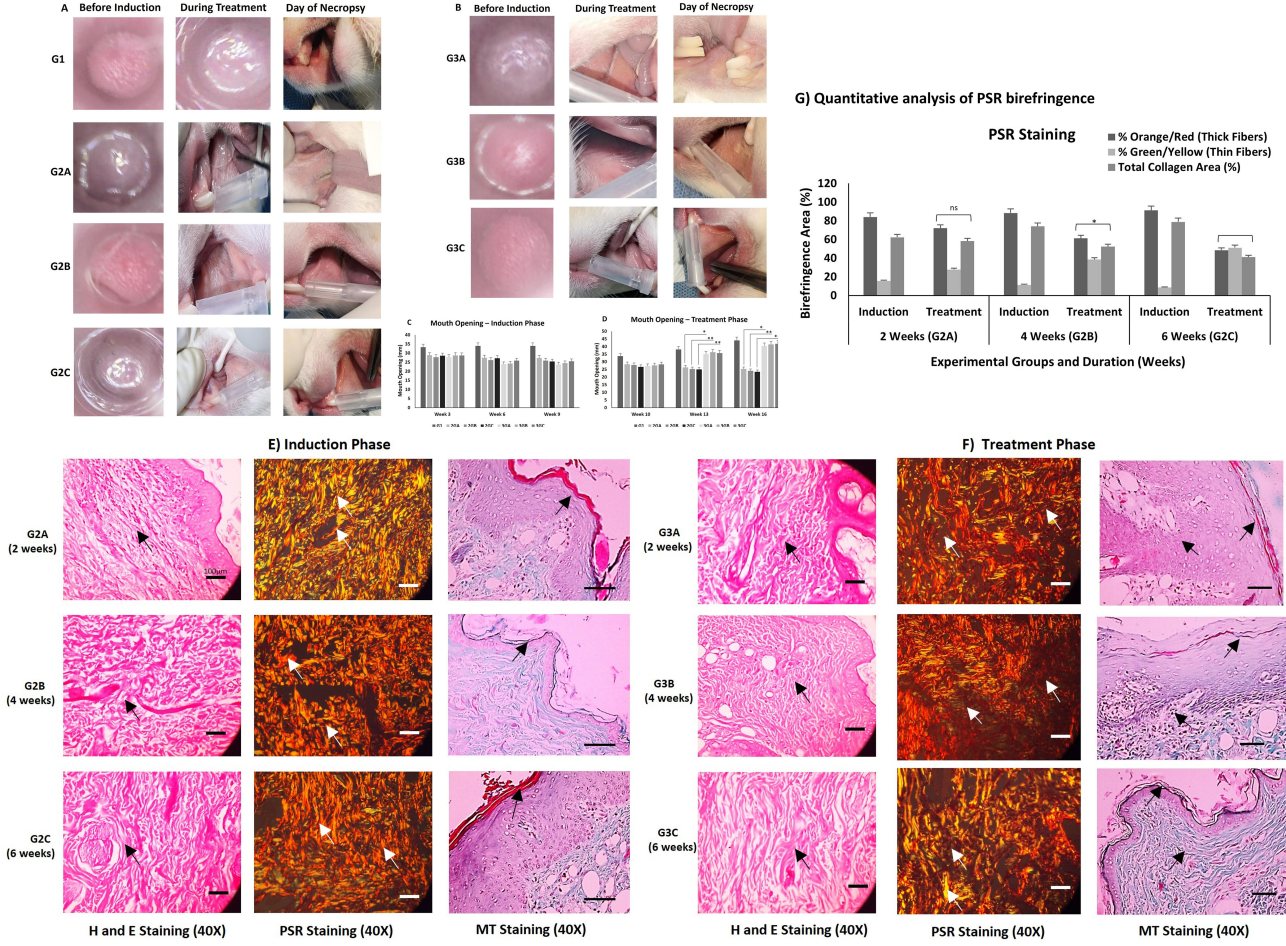
Effect of mucoadhesive buccal IBL patch on mouth opening and histopathological alterations in a rabbit model. **(A–D)** Weekly mouth opening measurements (mm) during the induction phase (Weeks 3, 6, and 9) and treatment phase (Weeks 10, 13, and 16). Groups include normal control (G1), PWD controls (G2A–G2C), and IBL patch–treated groups (G3A–G3C). Data are presented as mean ± SD. Statistical analysis was performed using one-way ANOVA with Tukey’s *post hoc* test (*p < 0.05, **p < 0.01, ***p < 0.001). **(E, F)** Representative photomicrographs (40X) of buccal mucosa stained with H&E, PSR, and MT during induction and treatment phases. Induction-phase tissues (G2A–G2C) show epithelial thinning, inflammatory infiltration (black arrows), and dense collagen deposition with orange/yellow birefringence (PSR) and intense blue collagen bundles (MT). Treatment-phase tissues (G3A–G3C) show restoration of epithelial structure, reduced inflammatory cells, and decreased collagen deposition. Scale bar as indicated. **(G)** Quantitative analysis of PSR birefringence showing percentage distribution of orange/red (thick) and green/yellow (thin) collagen fibers across induction and treatment groups. Statistical significance was determined by one way ANOVA followed by Tukey’s *post-hoc* test. *p < 0.05, **p < 0.01 compared to corresponding induction controls.

During the treatment phase, IBL-treated groups exhibited statistically significant improvements in mouth opening compared to corresponding PWD controls at multiple post-treatment time points (days 86, 93, 100, 108, and 114; p < 0.01) (**Figures 6A–D**). For instance, on day 86, the 2-week treatment group (G3A) showed greater mouth opening (33.64 ± 0.41 mm) compared with G2A (31.60 ± 1.86 mm). Similar trends were observed in the 4-week group (G3B vs. G2B: 36.30 ± 1.68 vs. 34.29 ± 2.77 mm) and the 6-week group (G3C vs. G2C: 35.77 ± 0.47 vs. 33.08 ± 1.88 mm), with the longest treatment duration showing the greatest functional improvement. Functional assessment in the present study was limited to measurement of mouth opening. Additional parameters such as mucosal suppleness or biomechanical properties were not evaluated and may provide further insight in future studies.

Necropsy revealed localized fibrotic changes in induced animals without gross abnormalities in major organs, suggesting that the observed effects were confined to the oral cavity. Although IBL treatment improved functional mouth opening and reduced lesion severity, residual mucosal alterations were still present at study completion, indicating partial response within the experimental timeframe. Previous animal models have demonstrated OSMF like features following areca nut exposure, including increased collagen deposition and genotoxic changes (79). Bromelain containing mucoadhesive systems have been reported to reduce inflammatory and collagen associated changes in experimental models (6, 51). Additionally, Takashima et al. described a 3D-printed apigenin-loaded mucoadhesive film evaluated in a 4NQO-induced rat oral carcinogenesis model (80). These reports support continued investigation of localized mucoadhesive platforms in experimental models of oral premalignant conditions.

The induction period of 78 days was required to consistently observe features such as blanching of the buccal mucosa and reduction in mouth opening, which served as functional indicators under the selected conditions. These changes developed gradually over time. The intervention period of up to 6 weeks, although shorter, was sufficient to observe measurable improvements, including partial restoration of mouth opening and reduction in mucosal blanching. The selected timeline was therefore considered appropriate for evaluating treatment-associated responses within the experimental framework.

### 3.9 Histopathological findings

Histopathological evaluation using H&E, PSR, and MT staining was performed to assess the development of fibrotic lesions, progression of fibrosis, and treatment-associated structural changes (81, 82).

#### 3.9.1 Induction phase

At 2 weeks (G2A), mild to moderate epithelial thinning and early collagen deposition were observed. PSR under polarized light showed predominantly green birefringence, consistent with thinner collagen fibers. By 4 weeks (G2B), epithelial thinning and inflammatory cell infiltration were more pronounced. PSR demonstrated mixed green and orange birefringence, indicating increased collagen fiber thickness and ECM accumulation. MT staining revealed enhanced collagen deposition extending toward adjacent muscle fibers. At 6 weeks (G2C), marked epithelial thinning and dense collagen deposition with hyalinized appearance were evident. Polarized PSR demonstrated predominantly orange birefringence, consistent with thicker and more densely organized collagen bundles (**Figure 6E**).

#### 3.9.2 Treatment phase

After 2 weeks of treatment (G3A), reduced inflammatory infiltration and comparatively lower collagen density were observed relative to induction controls. PSR showed an increased proportion of green birefringence compared with corresponding induction groups. At 4 weeks of treatment (G3B), collagen bundles appeared less compact, and MT staining demonstrated reduced collagen extension toward muscle fibers relative to induction-phase specimens. By 6 weeks of treatment (G3C), collagen density and hyalinization appeared reduced compared to untreated induction controls. The observed changes were predominantly indicative of generalized thinning and partial reorganization of collagen fibers rather than focal liquefaction of hyalinized collagen. Polarized PSR showed a relative reduction in orange/red birefringence with a corresponding presence of green fibers compared to induction groups; however, residual orange/red birefringence indicating persistent thick collagen fibers was still evident (**Figure 6F**). To support these observations, quantitative analysis of PSR birefringence was performed by measuring the percentage distribution of orange/red (thick collagen) and green/yellow (thin collagen) fibers. Quantitative analysis demonstrated a relative decrease in orange/red birefringence and a corresponding increase in green/yellow fibers in treatment groups compared to induction groups across the 2, 4, and 6 week time points (**Figure 6G**). However, the persistence of orange/red birefringence indicates incomplete remodeling of thick collagen fibers under the tested conditions.

#### 3.9.3 Comparative analysis of induction vs. treatment phases

During the induction phase, fibrosis progressed from 2 to 6 weeks, characterized by increasing epithelial thinning, inflammatory infiltration, and collagen accumulation. PSR staining demonstrated a gradual shift from predominantly green birefringence toward more intense orange birefringence, consistent with increasing collagen fiber thickness. MT staining correspondingly showed progressive collagen deposition within the subepithelial connective tissue. In contrast, treated groups exhibited comparatively lower collagen density and reduced inflammatory features at matched time points. PSR demonstrated a relative reduction in orange birefringence relative to induction controls, supported by quantitative analysis of birefringence distribution. Despite these changes, residual thick collagen fibers were evident, indicating that collagen remodeling was partial rather than complete. Examination of H&E-stained sections at 40× magnification allowed visualization of the epithelial–connective tissue interface, as indicated by annotated regions. No distinct focal liquefaction of hyalinized collagen was observed; rather, the changes were consistent with a generalized reduction in collagen density and partial reorganization. MT staining also showed decreased collagen accumulation (83, 84). Across the study duration, fibrosis severity increased during the induction phase, confirming establishment of the experimental model. Treatment with the IBL patch was associated with comparatively lower fibrosis severity and inflammatory infiltration relative to PWD controls; however, histological features of fibrosis persisted, indicating partial resolution within the 6-week treatment period. The histological observations correspond with functional improvements in mouth opening measured during the treatment phase. These findings suggest modulation of collagen organization rather than complete reversal of fibrotic architecture within the study duration.

The present study primarily emphasizes the formulation foundation by evaluating the functional stability and activity of the multi-component patch. Parameters such as bromelain enzymatic activity were assessed to confirm retention of functional properties within the formulation. The selected cytokines, including IL-6, TNF-α, and MMP-2, were analyzed as markers associated with inflammatory response and extracellular matrix remodeling, relevant to the observed functional changes such as mouth opening.

The *in vitro* system employed in this study represents an acute inflammatory response in epithelial cells and does not recapitulate the chronic, fibroblast-driven fibrosis characteristic of advanced oral submucous fibrosis. Markers such as IL-6 and TNF-α reflect early inflammatory responses that may contribute to downstream tissue alterations but do not directly correspond to the dense juxta-epithelial hyalinization observed in later stages of the disease. Accordingly, the *in vitro* findings are interpreted as indicative of cytokine-associated inflammatory changes, while the *in vivo* observations provide complementary insight into tissue-level remodeling.

Compared with conventional oral formulations, the IBL patch enables localized delivery with sustained mucosal contact through a mucoadhesive polymeric matrix. Multi-component buccal systems incorporating enzymatic, keratolytic, and permeation-associated agents within a single platform remain relatively limited (85, 86). In the present formulation, bromelain was evaluated as the primary enzymatic component for stability and collagenolytic activity, while isotretinoin and limonene were included as complementary agents with reported roles in epithelial regulation and inflammatory modulation. The combined incorporation of these agents within a polymer matrix provides a basis for coordinated local exposure under experimental conditions (7, 10, 14). Within the scope of the stability assessment, bromelain activity was retained over the 6-month evaluation period, suggesting that the formulation environment did not adversely affect enzymatic stability under the tested conditions. However, specific interactions or potential synergistic or antagonistic effects between individual components were not evaluated and require further investigation.

The polymer composition (EC, DBP, SALG, SCMC, and HPMC) was selected to support mechanical integrity, controlled release, and mucoadhesion while maintaining biological activity. Observed effects on cellular proliferation, migration, and extracellular matrix-associated processes suggest that incorporation within the matrix did not adversely affect functional activity under the tested conditions. Similar multi-component mucoadhesive systems have been reported previously, supporting the feasibility of localized oral delivery approaches (18, 21).

The findings of this study suggest that the IBL patch is associated with changes in cytokine expression patterns relevant to inflammation and fibrosis. Reduced expression of IL-6 and TNF-α may reflect alterations in inflammatory signaling, while decreased TGF-β expression suggests a potential influence on fibrosis-associated processes linked to extracellular matrix accumulation. The combined presence of bromelain, limonene, and isotretinoin may contribute to these observations; however, their individual contributions were not specifically evaluated. The concurrent modulation of multiple cytokine markers indicates a potential effect on interconnected inflammatory and fibrotic pathways.

Despite these observations, several limitations should be considered. Direct evaluation of specific signaling pathways such as NF-κB or MAPK was not performed, and pharmacokinetic profiling and long-term safety assessment remains to be addressed. Future studies should include component specific analyses, pathway-level validation, and expanded *in vivo* investigations to further clarify the underlying mechanisms and translational relevance of this approach.

### 3.10 Study limitations and translational considerations

The study does not include single-component controls, limiting attribution of individual effects. Mechanistic pathway analysis was not performed. The *in vitro* and *in vivo* systems represent distinct biological contexts. Long-term safety and sustained effects were not evaluated. While bromelain stability was evaluated, the specific contributions of isotretinoin and limonene were not examined through dedicated molecular or protein-level assays, which could provide additional mechanistic insight.

A decline in enzymatic activity during storage highlights the need for further optimization to ensure formulation stability. Residual solvent analysis following acetone evaporation (ICH Q3C Class 3) will be necessary for regulatory compliance. The present study does not include evaluation of intracellular signaling intermediates such as NF-κB, SMAD, STAT, p-NF-κB, IκB, p-SMAD2/3, p-STAT3 pathways, and therefore mechanistic conclusions cannot be drawn. Although preliminary cytocompatibility findings suggest acceptable short-term safety under experimental conditions, additional studies assessing mucosal irritation, repeated exposure, immunogenicity, and chronic toxicity are required prior to translational progression. CCl_4_ is a non-specific fibrotic agent and does not replicate areca nut-induced OSMF. It was selected for its ability to induce reproducible fibrotic and inflammatory changes under controlled conditions. The *in vitro* epithelial model and the *in vivo* fibrosis model represent distinct biological systems, and the findings are interpreted as complementary observations rather than directly equivalent outcomes.

### 3.11 Comparative perspective

Current management strategies for Oral Squamous Cell Carcinoma primarily focus on tumor reduction through surgery, chemotherapy, and radiotherapy. The IBL patch is not intended as a replacement for these approaches but represents a localized biomaterial-based delivery platform designed to provide sustained exposure within oral tissues under experimental conditions. Observed changes in cellular proliferation and migration suggest potential adjunctive relevance; however, these findings remain exploratory. Future work should prioritize long-term *in vivo* validation, pharmacokinetic evaluation, molecular pathway analysis, and stability optimization. Early phase safety assessment will be essential to determine translational feasibility. Overall, the study supports the feasibility of a multi-component mucoadhesive platform for localized delivery of bioactive agents, while further investigation is required to define its clinical relevance.

## 4 Conclusions

In summary, this study reports alterations in cytokine associated inflammatory and fibrotic markers following treatment in experimental models relevant to OSMF. The findings highlight describes changes in cytokine-associated markers and extracellular matrix responses under experimental conditions that may contribute to inflammatory and fibrotic processes, as observed in LPS induced acute inflammatory *in vitro* systems and *in vivo* chemically induced experimental fibrosis model. The formulation was associated with reduced IL-6, TNF-α, and TGF-β levels, along with regulation of IL-2 and MMP-2, suggesting an effect on inflammatory changes and extracellular matrix remodeling. These results indicate that a multi-component, biomaterial-based approach may influence interconnected inflammatory and fibrotic pathways within the oral microenvironment. The findings should be interpreted as preliminary observations supporting further investigation into formulation performance and biological activity. While the study is exploratory and does not establish direct mechanistic or clinical causality, the observations may inform further investigation into cytokine signaling mechanisms in chronic inflammatory conditions such as OSMF. Further studies are needed to delineate component specific effects, validate underlying signaling mechanisms, and assess long term safety and clinical applicability.

## Data Availability

The original contributions presented in the study are included in the article/**Supplementary Material**. Further inquiries can be directed to the corresponding authors.

## Supplementary material

Supplementary Figure 1 Drug content uniformity of IBL patch, demonstrating consistent drug distribution across all samples. Data are presented as mean ± SEM (n=3).

Supplementary Figure 2 IBL patch drug release performance at different pH levels (pH 4, 7, and 10), as measured by UV-visible spectrophotometry (200–600 nm). The release profiles demonstrate pH-dependent release order and controlled, sustained drug delivery across all tested conditions. Data are presented as mean ± SEM (n=3).

Supplementary Figure 3 Hemolysis assay results for IBL and drug-free patches, showing negligible hemolytic activity compared to the negative control after 2 hours of incubation. Data are presented as mean ± SEM, n=3.

Supplementary Figure 4 SDS-PAGE analysis showing time-dependent degradation of Matrigel by the bromelain-infused IBL patch. Progressive loss of collagen bands demonstrates effective proteolytic activity.

Supplementary Figure 5 *In vitro* cytotoxicity and functional evaluation of IBL patch and individual components in KB-3–1 and McCoy cells. **(A, B)** MTT assay showing dose-dependent effects of limonene, bromelain, isotretinoin, PWD, and IBL patch on KB-3–1 and McCoy cell viability after 24 h. **(C, D)** Colony formation assay showing representative images and quantification of KB-3–1 and McCoy cells treated with IBL patch (250, 500, and 750 µg/mL). **(E, F)** Wound healing assay showing representative images and quantification of KB-3–1 and McCoy cell migration at 0, 12, and 24 h following treatment. **(G, H)** AO/EtBr staining showing live (green) and apoptotic (orange/red) cells after treatment with PWD and increasing concentrations of IBL patch. Data are presented as mean ± SD (n = 3). *p < 0.05, **p < 0.01, ***p < 0.001 vs. control.

Supplementary Figure 6 Histopathological evaluation of treated and control oral tissues. Representative micrographs of oral tissues from control and treated groups at 10× and 40× magnifications. **(A, B)** H&E staining showing tissue morphology, epithelial thickness, keratinization, and stromal organization. **(C, D)** PSR staining illustrating collagen fiber distribution and density. **(E, F)** MT staining showing collagen deposition within the stromal region. **(G)** Semi-quantitative analysis of PSR birefringence showing percentage distribution of orange/red (thick/mature) and green/yellow (thin/immature) collagen fibers, along with total collagen area. *p < 0.05 vs. control. **(K)**, keratin layer; **(E)**, epithelium; **(D)**, dermis/connective tissue. Scale bars as indicated.

Supplementary Table 1a List of formulations used for standardization of IBL patch.

Supplementary Table 1b Standardization of mucoadhesive buccal patch with different formulations of IBL drugs.

Supplementary Table 2 Primers used for qPCR analysis.

Supplementary Table 3 Experimental grouping and treatment schedule for chemically induced rabbit oral inflammation model induction and mucoadhesive buccal patch intervention.

Supplementary Table 4 Swelling index (%) at one-hour intervals for: Sample 1: Mucoadhesive patch without drug (PWD). Sample 2: Mucoadhesive patch containing 0.05% Isotretinoin, 1% Bromelain, and 1% Limonene. Data are presented as mean ± SEM (n=3).
