# Supplementary material for: Evaluation of a multi-component mucoadhesive buccal patch: cytokine-associated inflammatory responses and tissue remodeling in experimental models
Source: Front Immunol. 2026 May 28;17:1847576. doi: 10.3389/fimmu.2026.1847576 (PMC13252781; doi:10.3389/fimmu.2026.1847576)
Supplement: Supplementary Figure 1 — Drug content uniformity of IBL patch, demonstrating consistent drug distribution across all samples. Data are presented as mean ± SEM (n=3). [file DataSheet1.docx]

**Supplementary Figures**


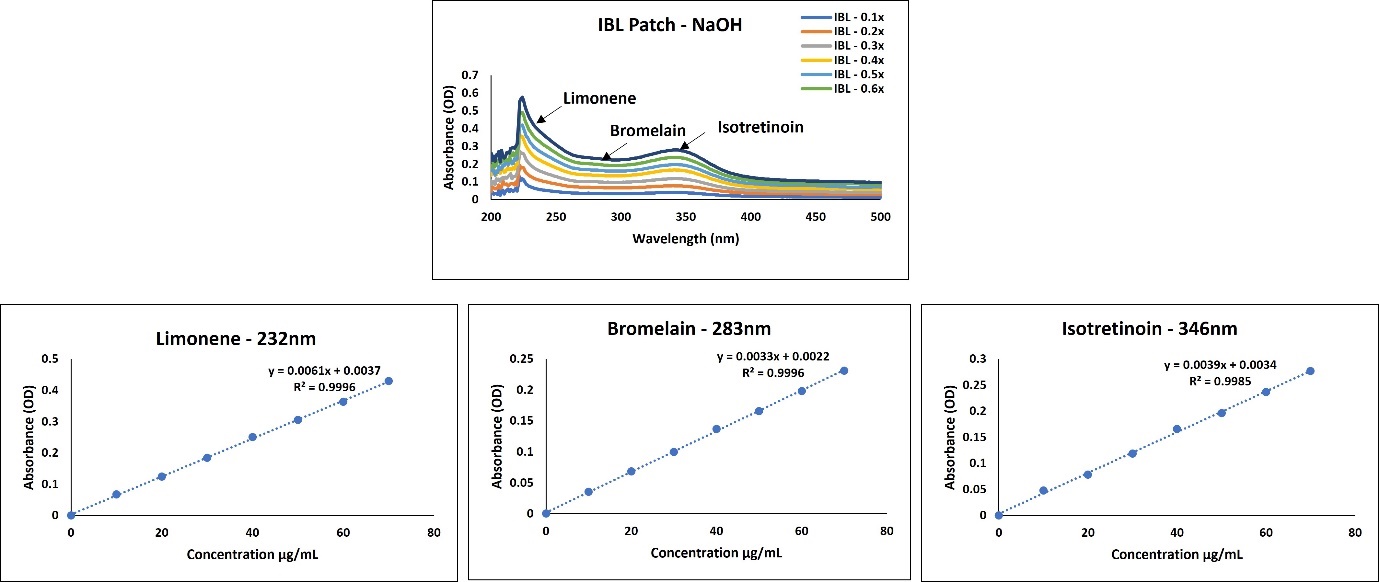


**Fig. S1.** Drug content uniformity of IBL patch, demonstrating consistent drug distribution across all samples. Data are presented as mean ± SEM (n=3).


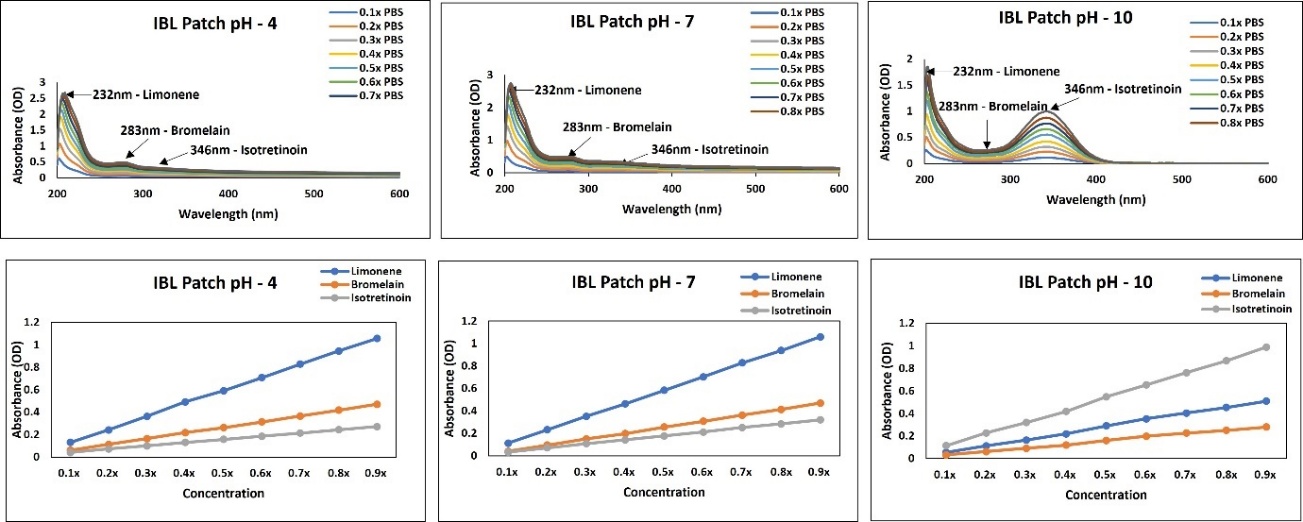


**Fig. S2.** IBL patch drug release performance at different pH levels (pH 4, 7, and 10), as measured by UV-visible spectrophotometry (200–600 nm). The release profiles demonstrate pH-dependent release order and controlled, sustained drug delivery across all tested conditions. Data are presented as mean ± SEM (n=3).


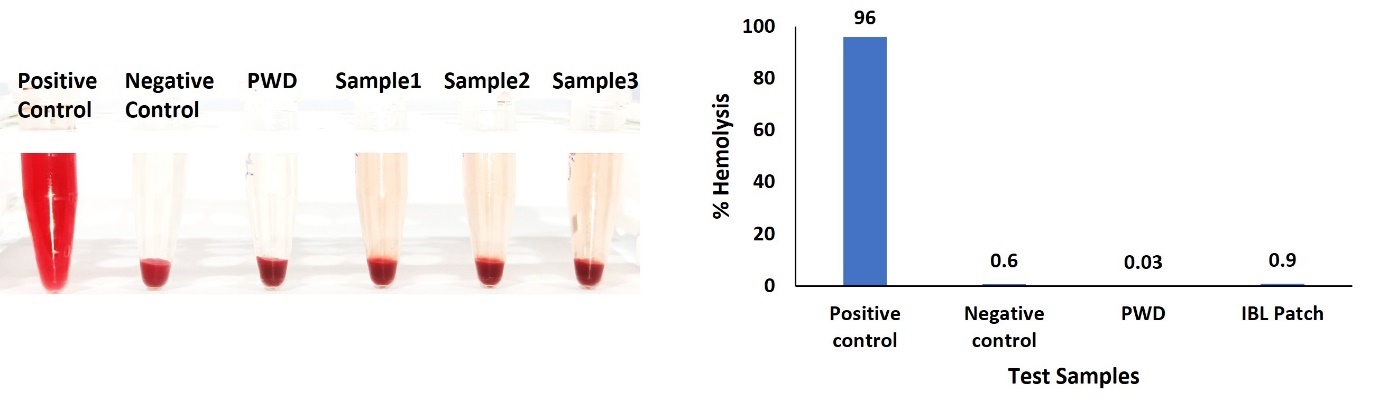


**Fig. S3.** Hemolysis assay results for IBL and drug-free patches, showing negligible hemolytic activity compared to the negative control after 2 hours of incubation. Data represented as mean±SEM, n=3.


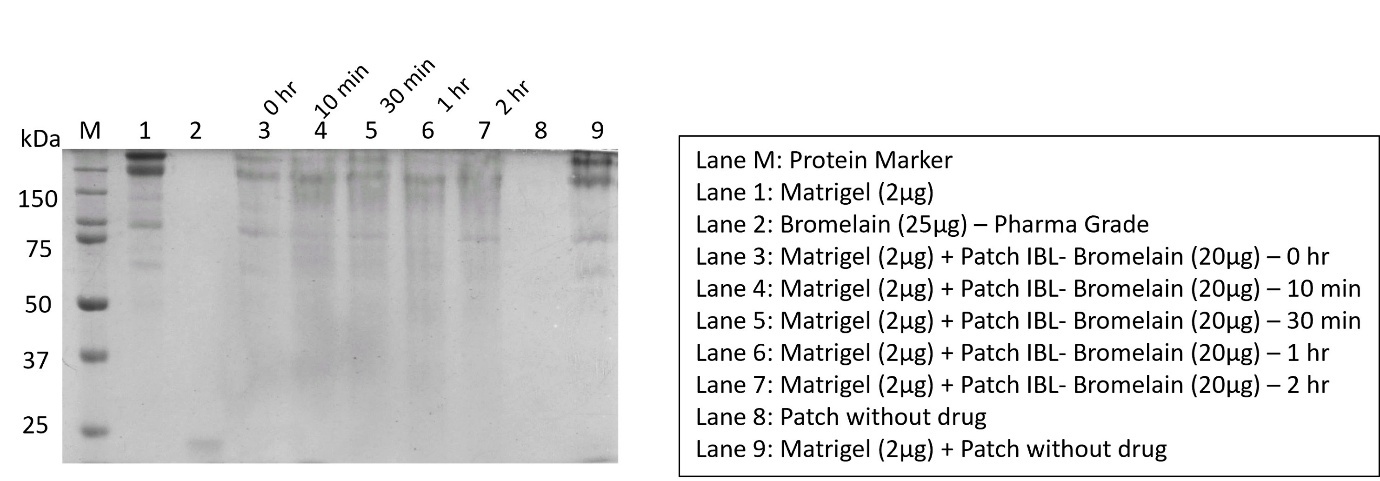


**Fig.** **S4.** SDS-PAGE analysis showing time-dependent degradation of Matrigel by the bromelain-infused IBL patch. Progressive loss of collagen bands demonstrates effective proteolytic activity.


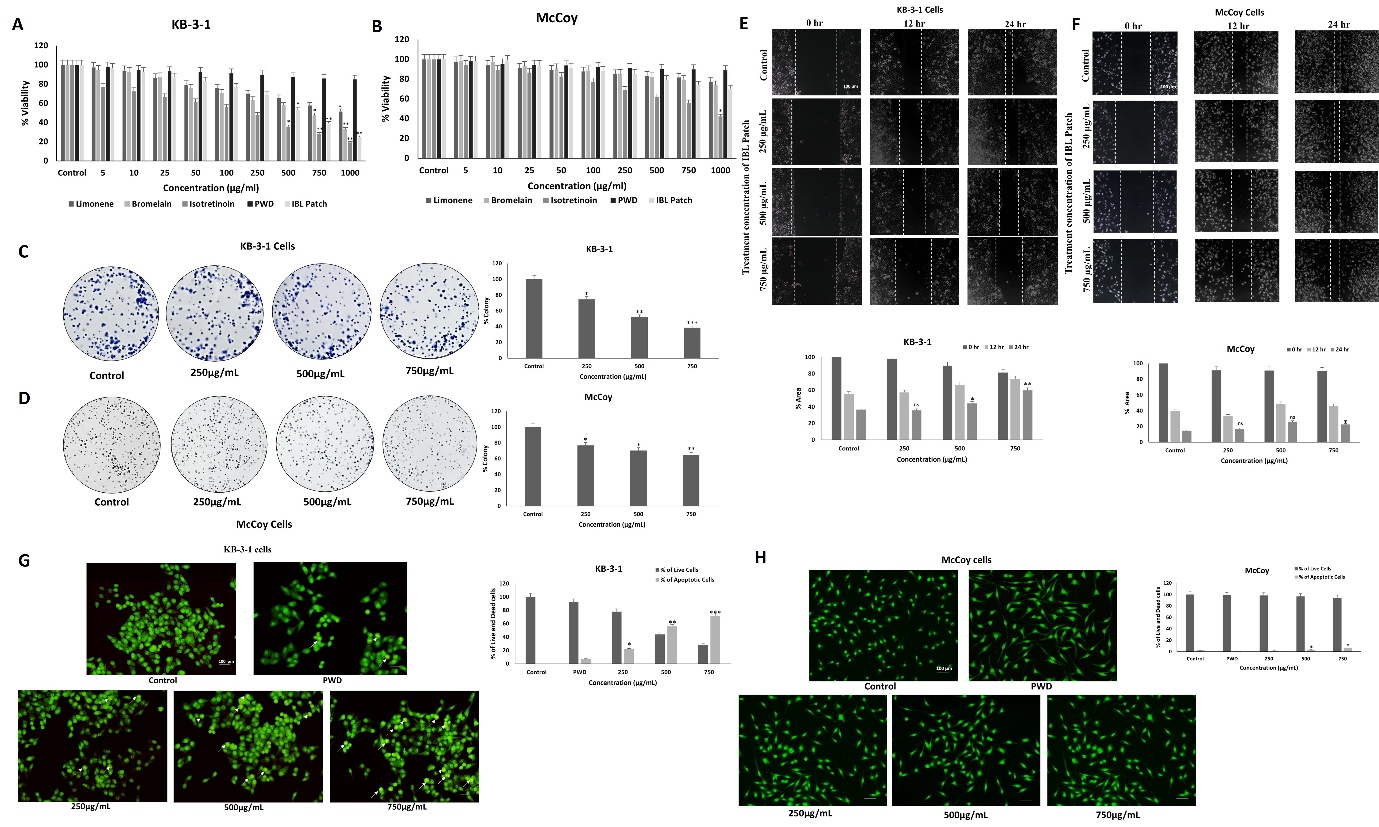


**Fig. S5.** In vitro cytotoxicity and functional evaluation of IBL patch and individual components in KB-3-1 and McCoy cells. (A–B) MTT assay showing dose-dependent effects of limonene, bromelain, isotretinoin, PWD, and IBL patch on KB-3-1 and McCoy cell viability after 24 h.
(C–D) Colony formation assay showing representative images and quantification of KB-3-1 and McCoy cells treated with IBL patch (250, 500, and 750 µg/mL).
(E–F) Wound healing assay showing representative images and quantification of KB-3-1 and McCoy cell migration at 0, 12, and 24 h following treatment.
(G–H) AO/EtBr staining showing live (green) and apoptotic (orange/red) cells after treatment with PWD and increasing concentrations of IBL patch. Data are presented as mean ± SD (n = 3). *p < 0.05, **p < 0.01, ***p < 0.001 vs. control.


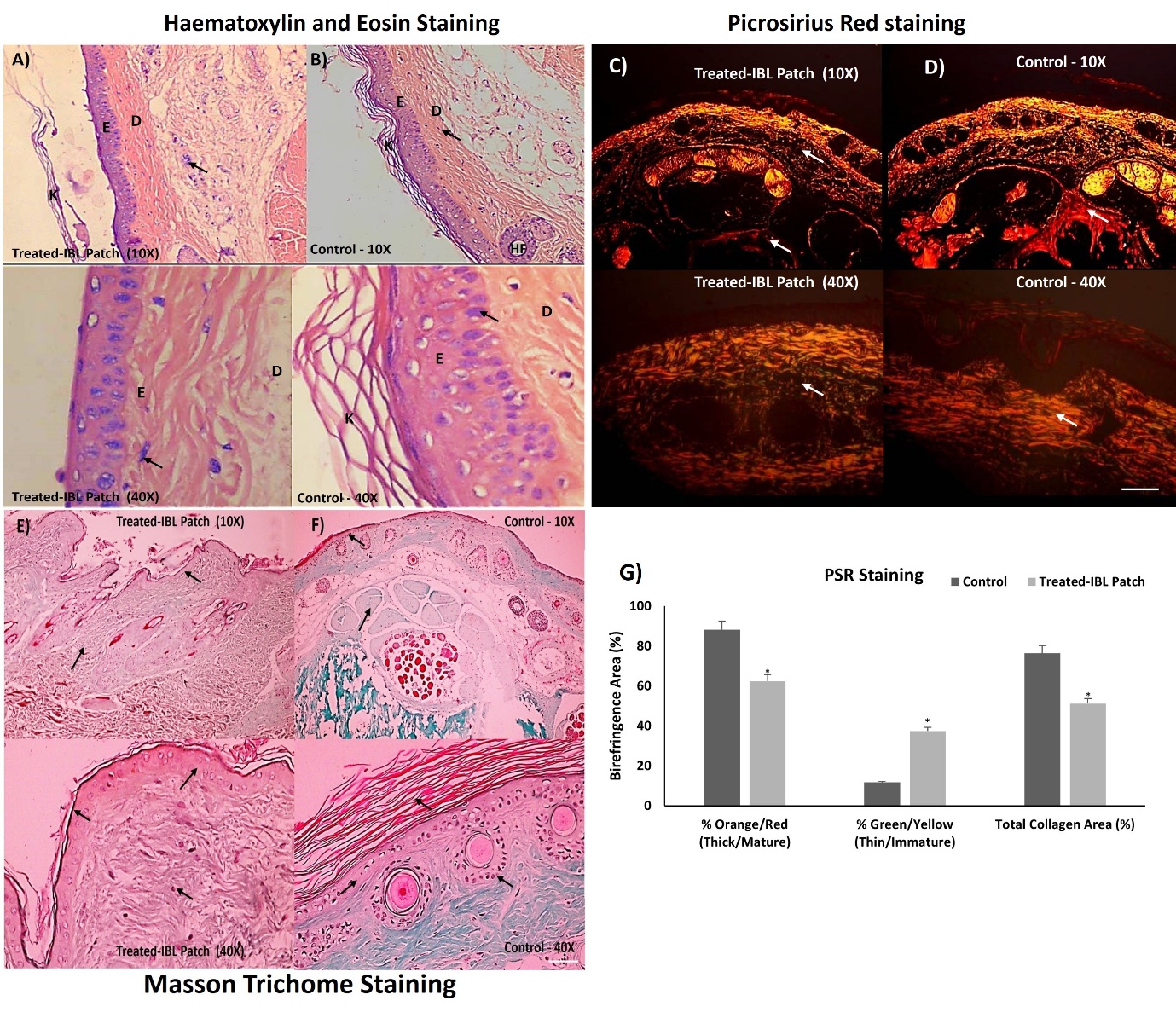


**Fig. S6.** Histopathological evaluation of treated and control oral tissues. Representative micrographs of oral tissues from control and treated groups at 10× and 40× magnifications.
(A, B) H&E staining showing tissue morphology, epithelial thickness, keratinization, and stromal organization. (C, D) PSR staining illustrating collagen fiber distribution and density.
(E, F) MT staining showing collagen deposition within the stromal region. (G) Semi-quantitative analysis of PSR birefringence showing percentage distribution of orange/red (thick/mature) and green/yellow (thin/immature) collagen fibers, along with total collagen area. *p < 0.05 vs. control. K – keratin layer; E – epithelium; D – dermis/connective tissue. Scale bars as indicated.

**Supplementary Tables**

**Table S1.** List of formulations used for standardization of IBL Patch

| **Formulations** | **SALG (%)** | **SCMC (%)** | **HPMC**  **(%)** | **Isotretinoin**  **(%)** | **Bromelain**  **(%)** | **Limonene**  **(%)** |
| --- | --- | --- | --- | --- | --- | --- |
| F1 | 1 | 3 | 2 | 0.5 - Water | 1 | 5 |
| F2 | 1 | 3 | 2 | 0.5 - Acetone | 1 | 5 |
| F3 | 1 | 3 | 2 | 0.5 - Water | 2 | 5 |
| F4 | 1 | 3 | 2 | 0.5 - Acetone | 2 | 5 |
| F5 | 1 | 3 | 2 | 0.3 - Water | 1 | 5 |
| F6 | 1 | 3 | 2 | 0.3 - Water | 2 | 5 |
| F7 | 1 | 2.5 | 1.5 | 0.05 - Water | 1 | 1 |
| F8 | 1 | 2 | 1 | 0.05 - Water | 1 | 1 |

**Table** **S1b.** Standardization of mucoadhesive buccal patch with different formulations of IBL drugs

| **Formulations** | **Result** | **Inference** |
| --- | --- | --- |
| **F1** | 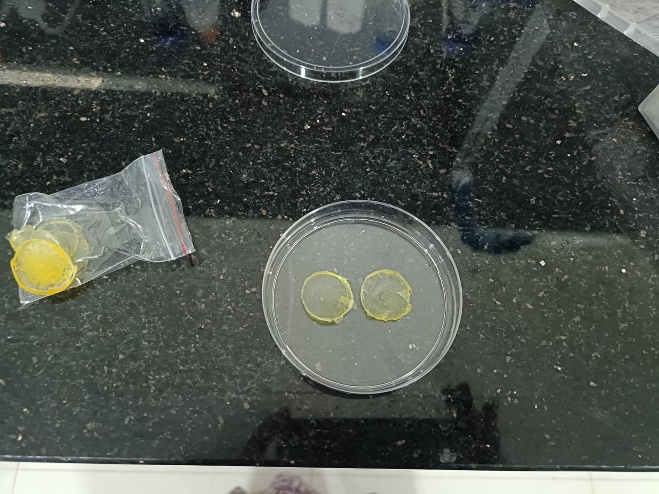 | **Very thin** and **brittle**. |
| **F2** | 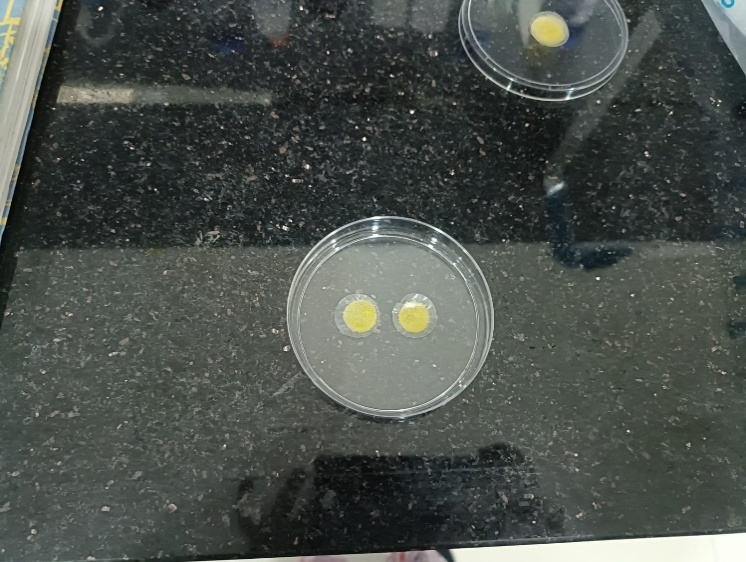 | **Thin film**, but the drugs were concentrated in the center, leading to non-uniform distribution. |
| **F3** | 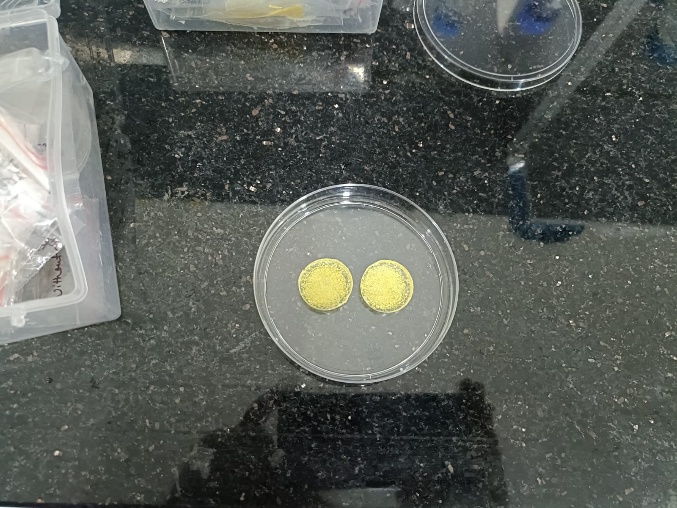 | **Thick and rigid**, with an uneven surface and lack of transparency. No peeling ability. |
| **F4** | 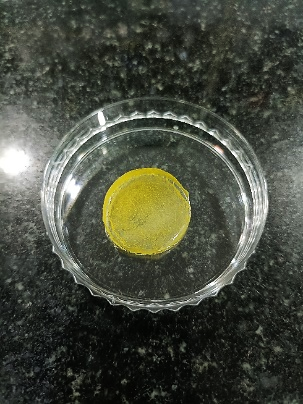 | **Thin and flexible**, but the drugs were not uniformly dissolved. |
| **F5** | 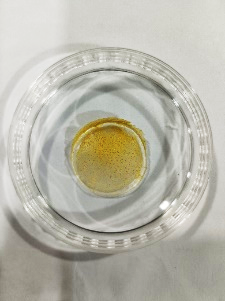 | **Thin and brittle**, with uneven drug distribution. |
| **F6** | 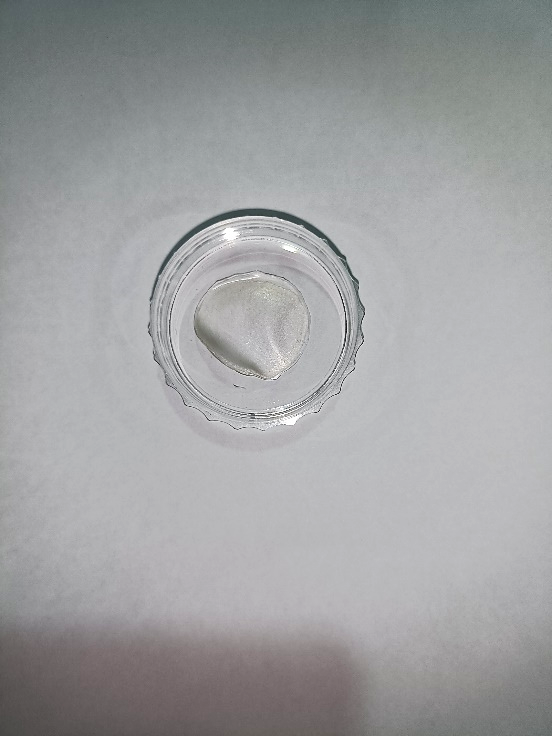 | **Thick and rigid**, with an uneven surface, lack of transparency, and no peeling ability. |
| **F7** | 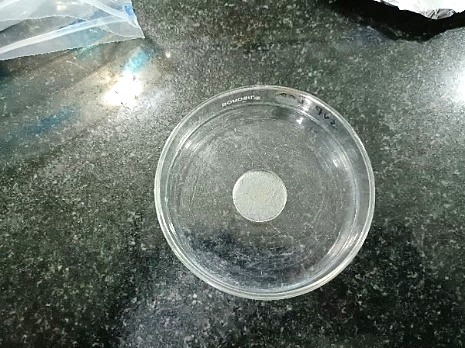 | **Thin, flexible, and transparent**, with good peeling ability |
| **F8** | 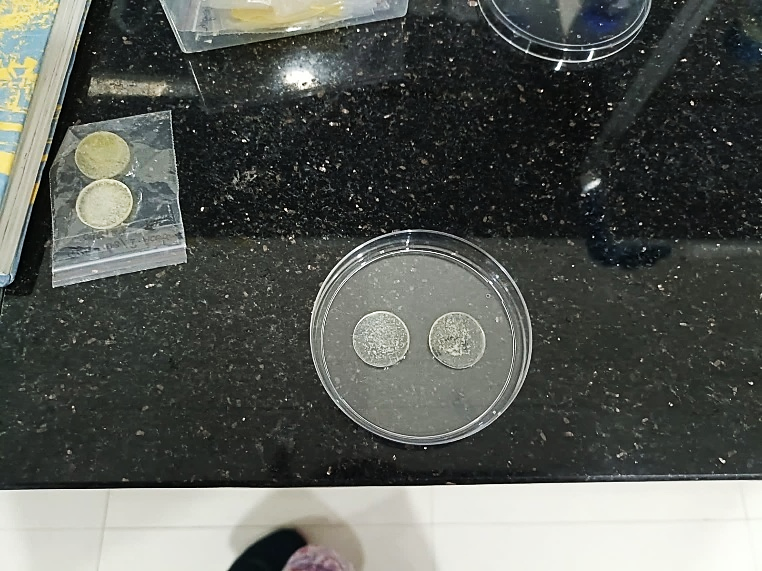 | **Thin, brittle** with powdered clumps |
| **Patch without drug** | 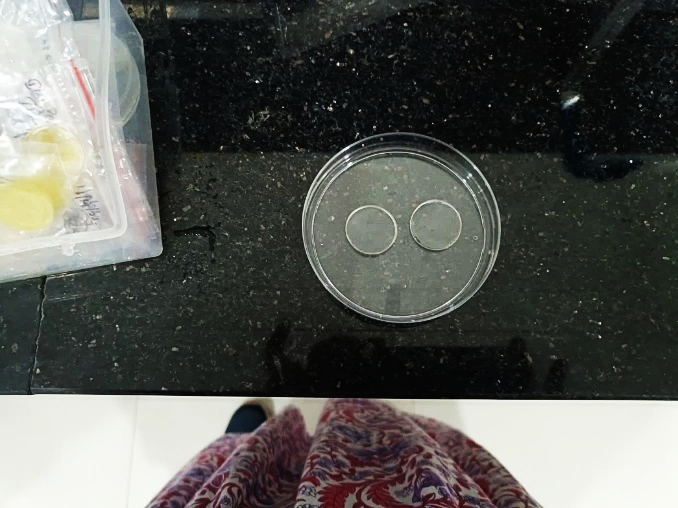 | **Thin and flexible**, with no polymer clumps. |
| **Only Backing Layer** | 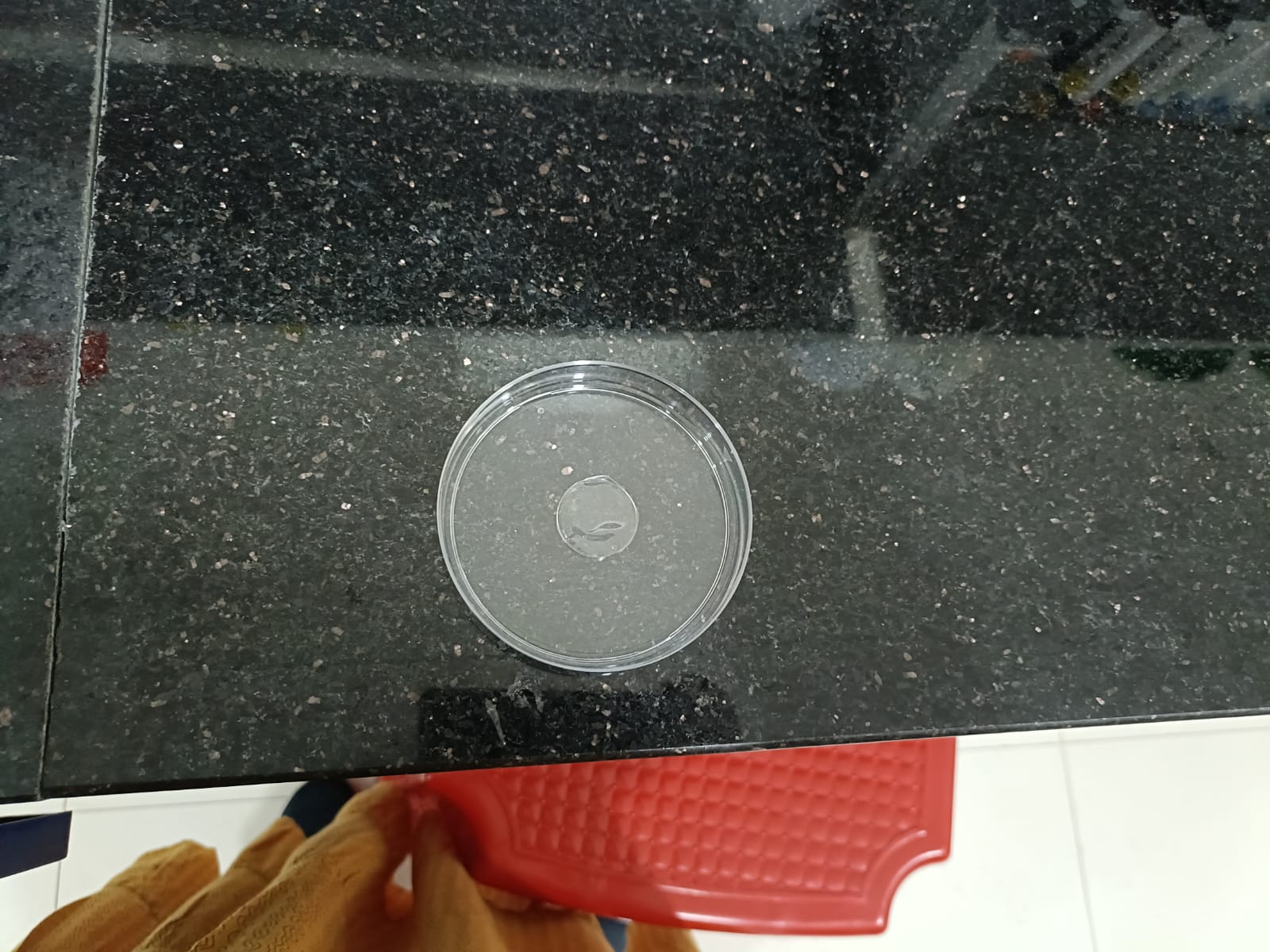 | **Thin** and **Transparent** |

**Table S2. Primers used for qPCR analysis**

| **Primers** | **Sequence** | **Base pairs** |
| --- | --- | --- |
| MMP2-F | CGCAGTGACGGAAAGATGTG | 20 |
| MMP2-R | AACAGGCTGTACCCTTGGTC | 20 |
| IL6-F | GGCACTGGCAGAAAACAACC | 20 |
| IL-6-R | TCACCAGGCAAGTCTCCTCA | 20 |
| IL2-F | TACATGCCCAAGAAGGCCAC | 20 |
| IL2-R | AGCACTTCCTCCAGAGGTTTG | 21 |
| TNFα-F | GCCCATGTTGTAGCAAACCC | 20 |
| TNFα-R | TGAGGTACAGGCCCTCTGAT | 20 |
| TGFβ1-F | CGTGGAGGGGAAATTGAGGG | 20 |
| TGFβ1-R | GCCGGTAGTGAACCCGTTGAT | 21 |
| GAPDH-F | GGAGCGAGATCCCTCCAAAAT | 20 |
| GAPDH-R | GGCTGTTGTCATACTTCTCATG | 20 |

**Table S3**. Experimental grouping and treatment schedule for chemically induced rabbit oral inflammation model induction and mucoadhesive buccal patch intervention

| **Groups** | **Number of Animals** | **Description** | **Induction Phase** | **Total Duration** | **Post-Induction Treatment Duration** | **Application Frequency** |
| --- | --- | --- | --- | --- | --- | --- |
| 1 | 3 | Control – Normal diet with water | - | 120 days | No treatment | - |
| 2A | 3 | CCl₄-induced oral fibrosis+ PWD (2 weeks) | 78 days | 92 days (13 weeks) | 2 weeks (14 days) | Twice per week |
| 2B | 3 | CCl₄-induced oral fibrosis+ PWD (4 weeks) | 78 days | 106 days (15weeks) | 4 weeks (28 days) | Twice per week |
| 2C | 3 | CCl₄-induced oral fibrosis+ PWD (6 weeks) | 78 days | 120 days (17 weeks) | 6 weeks (42 days) | Twice per week |
| 3A | 3 | CCl₄-induced oral fibrosis+ IBL Patch (2-weeks) | 78 days | 92 days (13 weeks) | 2 weeks (14 days) | Twice per week |
| 3B | 3 | CCl₄-induced oral fibrosis+ IBL Patch (4 weeks) | 78 days | 106 days (15 weeks) | 4 weeks (28 days) | Twice per week |
| 3C | 3 | CCl₄-induced oral fibrosis+ IBL Patch (6 weeks) | 78 days | 120 days (17 weeks) | 6 weeks (42 days) | Twice per week |

**Table S4.** Swelling index (%) at one-hour intervals for: Sample 1: Mucoadhesive patch without drug (PWD). Sample 2: Mucoadhesive patch containing 0.05% Isotretinoin, 1% Bromelain, and 1% Limonene. Data are presented as mean ± SEM (n=3).

| **Sample** | **Swelling Index (In Percentage) at one-hour Intervals** | | | | | | | | **Mean of Swelling Index %** |
| --- | --- | --- | --- | --- | --- | --- | --- | --- | --- |
|  | **1** | **2** | **3** | **4** | **5** | **6** | **7** | **8** |  |
| **1** | 26.14 | 35.14 | 13.00 | 11.50 | 4.37 | 3.04 | 3.80 | 3.57 | 12.57 |
| **2** | 17.93 | 42.69 | 38.93 | 22.12 | 11.84 | 6.48 | 3.85 | 2.34 | 18.27 |


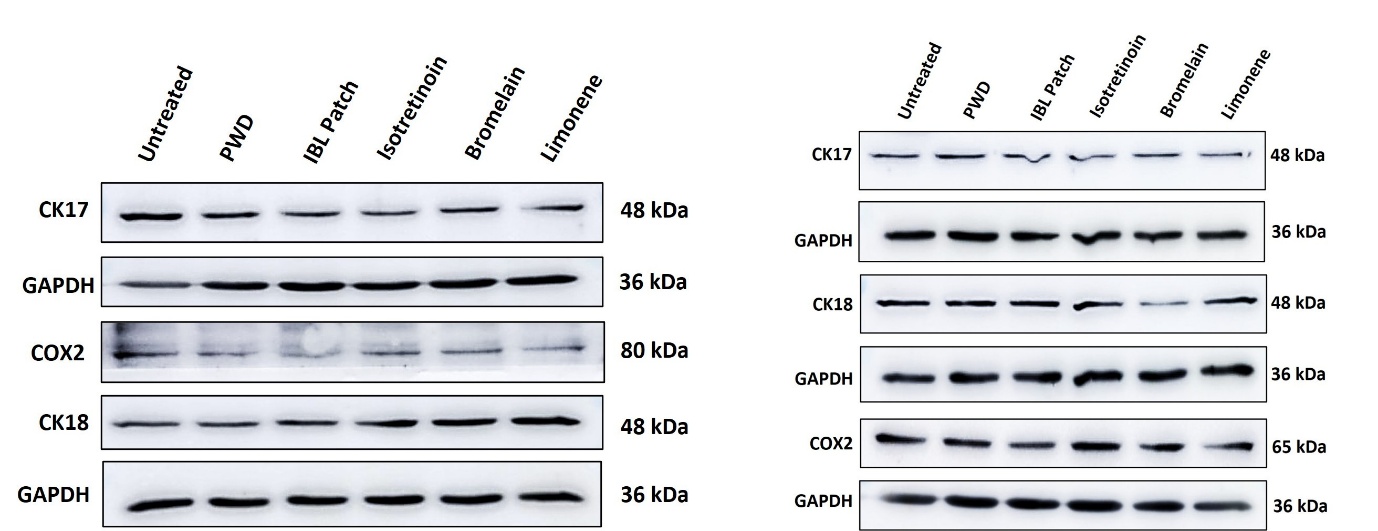


**Fig. 5F**. Western blots of 2^nd^ and 3^rd^ replications


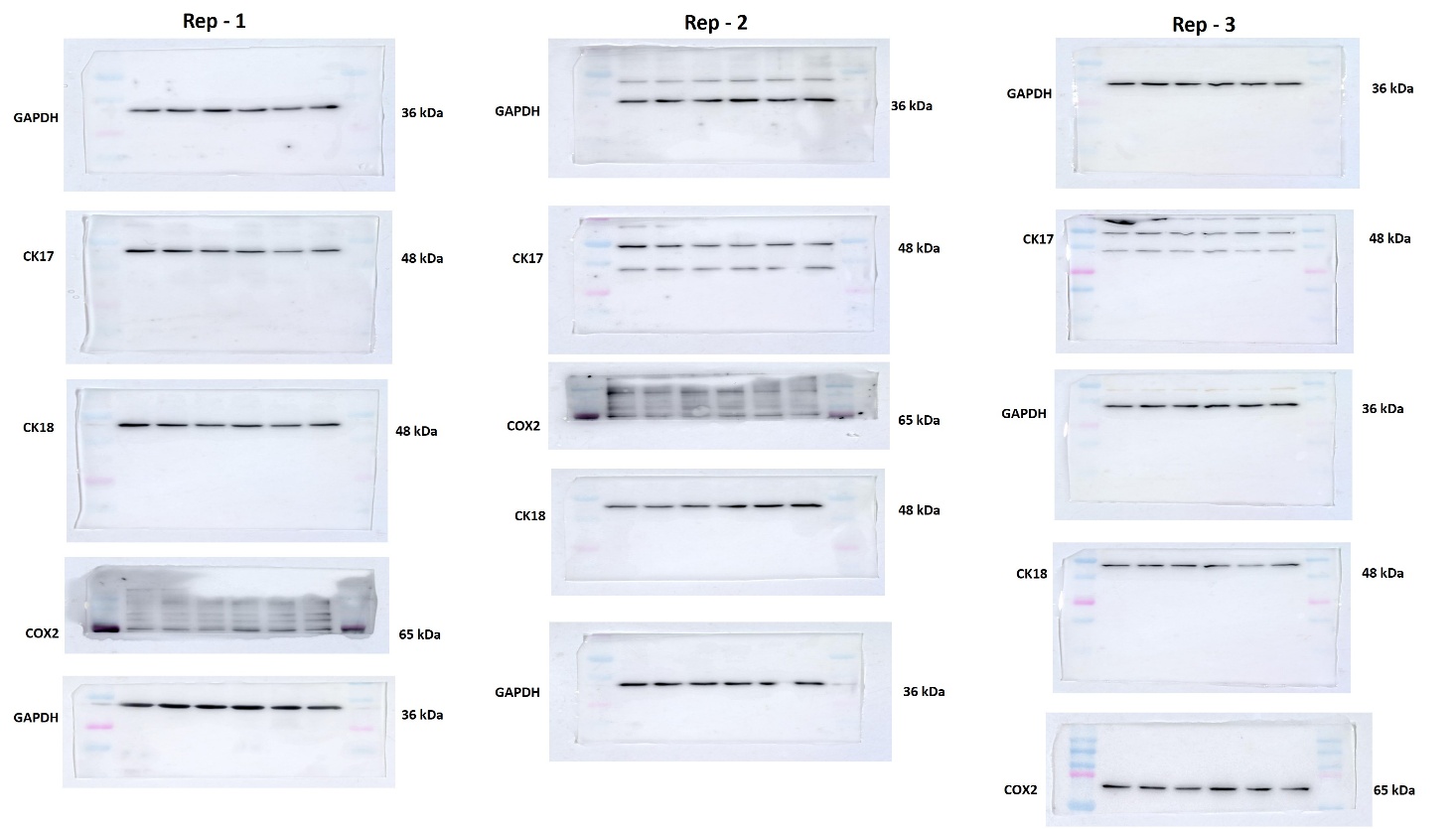


**Fig. 5F.** Western blots raw images of all three replications.
